# During the COVID-19 pandemic participants prefer settings with a face mask, no interaction and at a closer distance

**DOI:** 10.1038/s41598-022-16730-1

**Published:** 2022-07-27

**Authors:** K. Kühne, M. H. Fischer, M. A. Jeglinski-Mende

**Affiliations:** Cognitive Sciences Division, University of Potsdam, Karl‑Liebknecht‑Straße 24‑25, House 14, 14476 Potsdam, Germany

**Keywords:** Psychology, Human behaviour

## Abstract

Peripersonal space is the space surrounding our body, where multisensory integration of stimuli and action execution take place. The size of peripersonal space is flexible and subject to change by various personal and situational factors. The dynamic representation of our peripersonal space modulates our spatial behaviors towards other individuals. During the COVID-19 pandemic, this spatial behavior was modified by two further factors: social distancing and wearing a face mask. Evidence from offline and online studies on the impact of a face mask on pro-social behavior is mixed. In an attempt to clarify the role of face masks as pro-social or anti-social signals, 235 observers participated in the present online study. They watched pictures of two models standing at three different distances from each other (50, 90 and 150 cm), who were either wearing a face mask or not and were either interacting by initiating a hand shake or just standing still. The observers’ task was to classify the model by gender. Our results show that observers react fastest, and therefore show least avoidance, for the shortest distances (50 and 90 cm) but only when models wear a face mask and do not interact. Thus, our results document both pro- and anti-social consequences of face masks as a result of the complex interplay between social distancing and interactive behavior. Practical implications of these findings are discussed.

## Introduction

### The peripersonal and interpersonal space

The COVID-19 pandemic has changed our perception of the world, of ourselves and other people. Social distancing rules suggest that people should keep an interpersonal distance of at least 1 m (3 feet), as recommended by the World Health Organization. While Australia, China, Denmark, France, Hong Kong and Singapore stuck to this rule, several countries, such as India and Canada extended this distance to 2 m, Great Britain to 1 + m, but Germany, Switzerland, Belgium, Greece, Germany, Italy, Spain, Portugal and many other European countries recommended up to 1.5 m. The USA and South Korea prescribed distances of 1.8 m and 1.4 m, respectively. These demands of social distancing challenge our natural proxemic behaviors as well as our spatial estimation abilities.

Normally, a comfortable interpersonal distance to other individuals is a matter of cultural conventions^1^ and is further modulated by our cognitive representation of our peripersonal space—the space surrounding our body^2^. In particular, this space is influenced by our arm length as well as by emotional reactions triggered by another individual located within our peripersonal space^3^. That means that peripersonal space and interpersonal distance are closely related to each other and share common motor resources^4^. Peripersonal space describes reachability distance for objects while interpersonal distance refers to comfortable distance to others^5^. Moreover, both the peripersonal space and, as a consequence, the comfortable interpersonal distance increase after using a long tool, which is known as an enlargement effect^6^.We briefly review the two concepts of peripersonal space and interpersonal distance to motivate our study.

### The peripersonal space

Within peripersonal space we make use of action opportunities and escape danger. The term was coined by Rizolatti and colleagues in a seminal study that found specific neurons of a monkey activated by stimuli in the near-body space^7^. The major functions of peripersonal space are considered to be multisensory integration of stimuli and action preparation. While an approximate size of peripersonal space was earlier estimated as about 30–50 cm around the hands and 60 cm around the head^8^, recent studies bring evidence that it is rather flexible and can be modulated by different factors, such as pregnancy^9^, reward^10^, environment temperature^11^ and pressure^12^, tool use^13–15^, hormone administration^16^ and even cultural belonging^17^. Moreover, social factors, such as the presence of another individual, can modulate the size of peripersonal space^18^. Teneggi and colleagues^18^ demonstrated that the boundaries of peripersonal space shrank when participants faced another individual and merged when individuals behaved cooperatively. Especially sensitive to such situative influences is the peripersonal space in the area near the face^19^.

Thus, peripersonal space and interpersonal distance are interrelated and share common mechanisms of self-protection^5^. It was demonstrated that social information can modulate both in a similar way^3^. Several studies have investigated the relation between social distancing, comfortable interpersonal distance, peripersonal space boundaries and danger perception, as well as human approaching and distancing endeavors. More recently, the COVID-19 pandemic has had an impact on these relations, as the perception of danger has changed in the course of different pandemic waves and with the introduction of various safety regulations.

There is evidence that the presence of a threatening stimulus near the body alters the representation of peripersonal space by changing our perceived reaching range^20–22^. In terms of the COVID-19 pandemic, a face mask could be such a threatening stimulus because it signals a contamination risk, and enlarges our peripersonal space. On the other hand, in the context of the pandemic a mask might also be considered as a safety tool, thereby shrinking our peripersonal space as well as interpersonal distance^22,23^. It is of note that the modulation of peripersonal space in the context of the ongoing COVID-19 pandemic is most probably caused by the subjective perception of danger rather than by a real danger^24^. This perception, in its turn, can depend on multiple factors including one’s own infection history, anxiety, risks, beliefs about the disease, and perceived vulnerability, for example, due to age. Thus, it was often pointed out that the elderly are especially vulnerable to the infection.

A recent study, conducted in Switzerland in the beginning of the COVID-19 pandemic showed a reduction of peripersonal space due to social distancing^25^. The authors adapted a classical multisensory task that is normally used to measure peripersonal space. Participants had to respond as fast as possible to a tactile stimulation on their faces. In the visuo-tactile (multimodal) stimulation condition, an avatar was approaching them in virtual reality from different distances. In the only tactile (unimodal) stimulation condition they only had to respond to the stimulation on their faces. The measurements were done at three time points: in the Pre-Pandemic phase (June–July 2018), in the Pre-Lockdown phase (10 February–10 March 2020), and in the Post-Lockdown phase (10 June–25 July 2020). In all but the Post-Lockdown cohorts, multimodal reaction times were significantly faster than unimodal ones (a facilitation effect). Only in the Post-Lockdown Cohort, avatars at farther locations elicited less facilitation on tactile processing (measured as reaction times), thus demonstrating a reduction of peripersonal space representation. The authors explained this phenomenon by invoking the “freezing” mechanism in a situation of danger^26^. Other individuals do not trigger any anticipatory processing when at a far distance, but a stronger tactile processing when at potential contamination distance.

Sakuma and Ikeda^27^ found an unexpected increase in peripersonal space in a study with a masked face condition compared to an unmasked face condition. This suggests that the perceived interpersonal distance was shorter when the approaching face was unmasked rather than masked, which might reflect a wish to adopt a larger distance to an unmasked person, in order to keep safe.

### Comfortable interpersonal distance

A preferred interpersonal distance is calculated within the first 600 ms of a social encounter^28^. Interpersonal distance is subject to modulation by gender, age and the degree of cooperation^29^. According to the Equilibrium theory, comfortable interpersonal distance is a compromise of approach and avoidance forces^30^. In particular, comfortable interpersonal distance reduces in cooperative tasks, while interaction at larger distances causes discomfort^28^. On the contrary, social threat^31^, arousal^32^ and contamination danger^33–35^ increase comfortable interpersonal distance.

One of the first studies on effects of the COVID-19 pandemic on interpersonal distancing was conducted by Marchiori and colleagues^36^ in Italy. Using a self-made social-distancing belt, the researcher measured the distance people adopted to an unmasked person, as well as from a person wearing different types of masks and goggles. The results were paradoxical: The distance adapted to a masked person was larger than the distance adapted to an unmasked person. This finding can be accounted for by invoking danger avoidance behaviors; since at the beginning of the pandemic wearing a mask was still unusual and probably meant that the person might have contracted COVID-19 or was contagious. To the same conclusion came Seres and colleagues^37^ who measured the influence of wearing a face mask in public waiting lines both when masks were mandatory and when they were not. In both cases the mask increased the comfortable distance between persons.

On the whole, as an interview study in Germany showed, the comfortable interpersonal distance was larger during the COVID-19 pandemic than before, especially during the pandemic peaks^38^.

Contrary to these findings, a later online study conducted with French participants found that wearing a mask rather reduces the comfortable interpersonal distance because people with a mask were considered to be more trustworthy^23^. Participants had to state their preferred interpersonal distance when facing human-like characters that were either wearing a face mask or displaying a neutral, happy or angry facial expression. Interestingly, the comfortable distance was more reduced in participants infected with COVID-19. These findings were corroborated by an Italian study^39^, but only for males. For females, the crucial factor in choosing a distance to a face was its emotional expression. The female participants adopted a shorter distance to happy faces than to angry ones. There is further evidence of reduction of the comfortable interpersonal distance towards individuals wearing protective equipment and who were tested negative to COVID-19^40^. The latest study done in virtual reality also supported the evidence of a comfortable interpersonal distance reduction when either the participant and/or the virtual agent wore a mask^41^.

In general, most studies showed an increase of interpersonal distance as a reaction to the pandemic and social distancing, but its reduction when the interlocutor wore a mask. Little is known about peripersonal space in the pandemic, though. Table 1 gives an overview of the results of previous research.

**Table 1:** Overview of studies on peripersonal space and interpersonal distance.

Peripersonal space	Interpersonal distance
Serino et al.^25^**↓**	Welsch et al.^38^ **↑**
Sakuma and Ikeda^27^ **↑** (mask)	Cartaud et al.^23^ **↓** (mask)
	Kroczek et al.^41^ **↓** (mask)
	Calbi et al.^39^ **↓** (mask, only males)
	Lisi et al.^40^ **↓** (mask and gloves)
	Seres et al.^37^ **↑** (mask)
	Marchiori et al.^36^ **↑** (mask)

↑ Stands for increase, ↓ stands for decrease of peripersonal space and interpersonal distance, respectively.

While there are still protests against masks or social distancing^42^, it is crucial to understand how peripersonal space and, in connection to this, interpersonal distance vary with such factors as a face mask and social behavior, such as intention to interact, and how these factors might change our social interactions. To this end, it is important to relate the face mask to interpersonal space.

### The present study

Previous studies in the context of COVID-19 evaluated the comfortable interpersonal distance or peripersonal space with explicit measures^23,38,39^. Although explicit and implicit measures are often related^43,44^, this relationship is moderated by multiple factors such as self-presentation, evaluative strength or automaticity, and distinctiveness^45^. Self-presentation presupposes concerns about the objectivity of self-reports, strength deals with the potency and importance of the evaluation, while distinctiveness is perception of one’s evaluation as unique in comparison to others. These factors are especially critical when estimating comfortable interpersonal distances in the context of the COVID-19 pandemic in the following ways. First, self-presentation can be biased by the forced social distancing (either by adhering to it or by confronting it). Second, evaluative strength can be modulated by the automatically preferred social distance or by the automatically processed contamination danger. Third, distinctiveness can be challenged by individuals’ ability to estimate distances. Bearing in mind these considerations, we preferred an implicit measure in our study.

In an online experiment, participants had to judge the gender of depicted persons. The pictures presented to them showed two individuals standing in three different distances to each other, either interacting by initiating or conducting a handshake or not, and either wearing a face mask or not. Judgments were recorded via button presses and participants were instructed to respond fast.

### Predictions

In general, reaction times can be interpreted in two ways. On the one hand, shorter reaction times are known to reflect an approach tendency to positive stimuli while longer reaction times reflect avoiding tendencies (positivity bias^46^). For instance, faster reaction times have been observed to positive faces^46^ compared to negative ones. On the other hand, an opposite pattern of responses—the negativity bias^47^ and faster reaction times for negative stimuli—has been documented in several studies with negative compared to positive words^48^ or faces^49^.

In accordance with the studies by Cartaud and colleagues^23^, Calbi and colleagues^39^, Sakuma and Ikeda^27^ and Welsch and colleagues^38^, we predict that the further individuals are in the pictures, the faster participants’ reaction times will be (positivity bias) (H1). In addition, we predict faster reaction times for pictures with a face mask compared to pictures without a face mask (positivity bias) (H2). Participants will prefer pictures with face masks because they evaluate them in a positive way. Moreover, we predict slower reactions when there is social interaction compared to no interaction, again, due to a preference in not having social interaction and running the risk of being contaminated (positivity bias) (H3). Shaking hands is not appropriate in the times of the COVID-19 pandemic and should be avoided.

Thus, based on previous research we postulate that a face mask rather reduces interpersonal distance and, presumably, peripersonal space, by signaling safety and trust, while in general interpersonal distance has increased in accordance with the social distancing rules and danger perception.

Crucially, we predict an interaction effect of face mask and social-interaction in dependence of the distance between the individuals, with slowest reaction times at near and middle distances without a mask as an avoidance signature and with fast reaction times for larger distances with a face mask (H4).

However, due to the partially exploratory nature of the study, we cannot completely rule out the alternative pattern (negativity bias), with faster reaction times due to an avoidance intention of an interaction with a face mask and at a closer distance.

We also explore possible interactions with demographic variables as well as attitudes towards different aspects of COVID-19 pandemic (see “Measures”).

## Materials and methods

### The sample

We determined the a priori sample size based on data simulation in ANOVA_exact app (https://arcstats.io/shiny/anova-exact/) considering an optimal level of statistical power (80% with an α-level of 5%). The recommended minimum sample size was rounded to 230 subjects. The simulation report can be found on the OSF under https://osf.io/jqdx8/.

In our calculations of the means and standard deviations we relied on a study with reaction times in cognitive experiments by Semmelmann and Weigelt^50^ as well as our own previous experiments on Gorilla software. The within-subject correlation was taken from similar reaction time studies^51^.

Although the critical three-way interaction showed 78% power, we perceived it as a satisfactory approximation to the desired power of 80%, bearing in mind recruiting difficulties during the pandemic. Since the power of the main effects and the critical interaction was satisfactory, we left several two-way effects being potentially underpowered.

### Participants

Two hundred and thirty-five participants (210 native German speakers, 25 participants with another native language) participated in the online study (38 males, 194 females, 3 divers; mean age = 24 years, *SD* = 5.8; 14% with university qualification (bachelor and above)). The participants were recruited via the participant recruitment system SONA at the University of Potsdam during five months between January 2021 and May 2021. This was the peak and the end of the third pandemic wave in Germany, when people were already used to social distancing and wearing masks.

### Procedure

#### The experiment and task

The experiment was created and hosted with Gorilla^52^. Participants were told that the study dealt with recognizing people in pictures. They were instructed to evaluate the people’s gender using either the P or the Q key on their keyboard. In one block they had to respond if at least one of the individuals in the picture was a man and in the other block, a woman. For example, in one block, if one of the individuals was a man, they had to press the P key and if there were no men in the picture, they had to press the Q key. The blocks were counterbalanced within participants. The pictures were shown in an individually randomized order and remained on the screen until response.

#### Stimuli

The pictures were self-created using licensed and license-free images from internet databases. For licensed pictures, we used databases Adobestock^53^, Shutterstock^54^, and 123rf^55^. Also, we used the database Pixabay^56^ for freely available pictures. The stimuli were created by cutting the background and combining the displayed persons. For this purpose, the picture manipulation programs GIMP^57^ and MS Power Point were used. Pictures of persons were combined in such a manner that the distance between them would be approximately 50 (near), 90 (middle) or 150 (far) cm in real life. To create these distances, body size of the depicted persons was used as measure, and they were placed in front of each other in the according scales, normalizing the distance by apparent body height.

**Figure 1:**
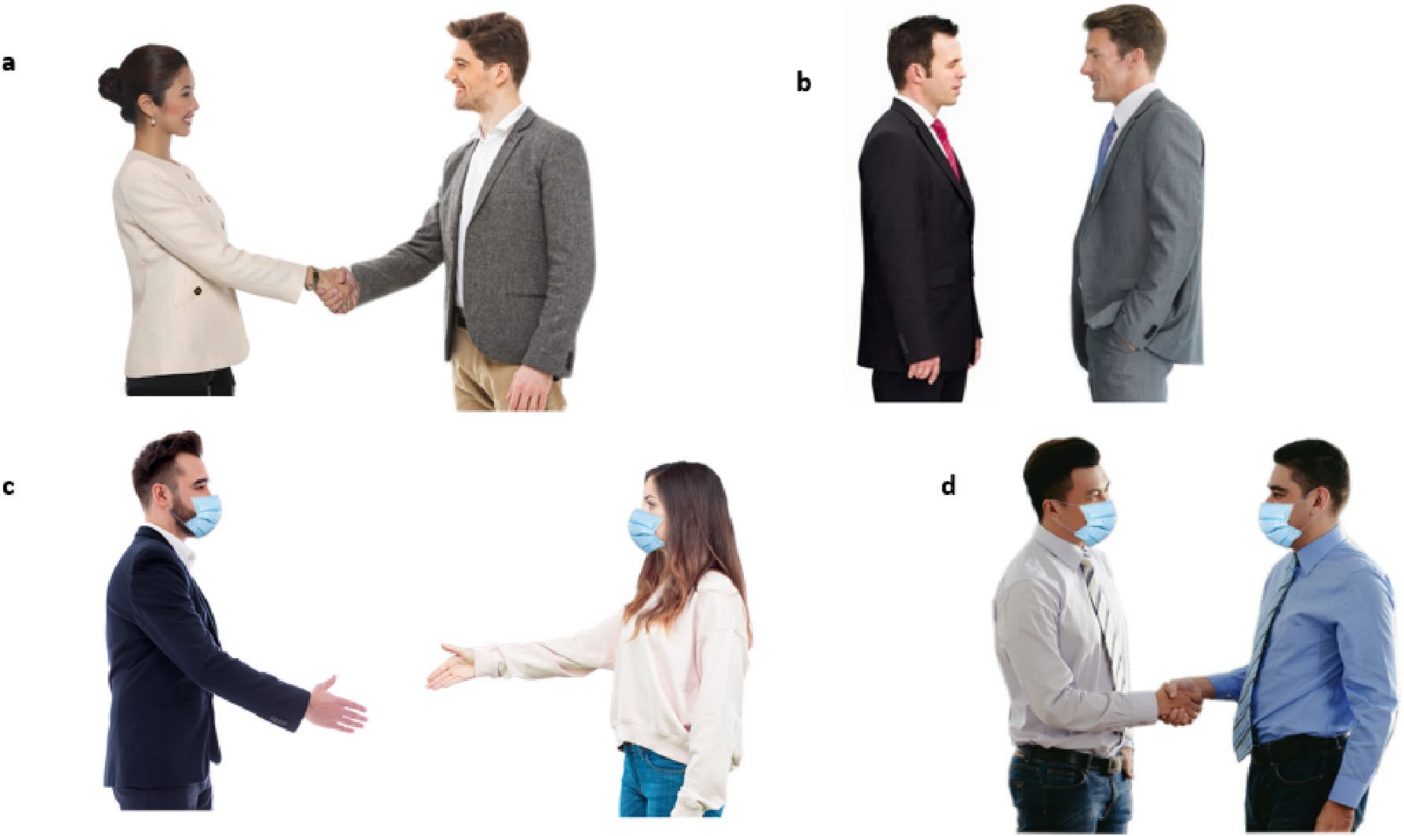
Sample stimuli. Panel (**a**) female-male, 90 cm, interaction, no mask. Panel (**b**) male-male, 50 cm, no interaction, no mask; Panel (**c**) male–female, 150 cm, interaction, mask. Panel (**d**) male-male, 50 cm, interaction, mask.

Also, interaction of depicted persons was manipulated, i.e. shaking of hands or no interaction. Moreover, the persons in the picture either wore no face mask or a face mask. Face masks were put into the pictures with the picture manipulation program, so that all persons wore the same mask (light-blue standard face mask). Furthermore, different pairs were created: female-female, male-male, female-male (female on the left), male–female (female on the right). We only used pictures of persons with rather neutral or positive facial expressions and in business-like or neutral clothes. 48 different stimuli were used (3 levels of distance (near/middle/far) × 2 levels of interaction (shaking hands/no shaking hands) × 2 levels of face mask (face mask/no face mask) × 4 levels of gender (female-female/male-male/female-male/male–female) = 48 stimuli). Figure 1 demonstrates sample pictures. See Supplementary Materials on https://osf.io/jqdx8/ for a full list of stimuli.

#### Measures

The main dependent variable was reaction time.

After the experiment participants answered demographic questions about their gender, age, native language, education, as well as the Edinburgh handedness inventory–short form^58^.

Additionally, they were asked several questions about the COVID-19 pandemic: 1. How dangerous do you consider COVID-19? (On a Likert scale from 1 = completely undangerous to 5 = very dangerous) 2. Do you consider COVID-19 as a danger for other people? (On a Likert scale from 1 = completely undangerous to 5 = very dangerous) 3. Do you consider COVID-19 as a danger for young people? (On a Likert scale from 1 = completely undangerous to 5 = very dangerous) 4. Do you consider COVID-19 as a danger for old people? (On a Likert scale from 1 = completely undangerous to 5 = very dangerous) 5. Do you think that wearing a face mask can protect from COVID-19? (On a Likert scale from 1 = no protection at all to 5 = very good protection) 6. Do you live together with someone who has risk factors for COVID-19? (Yes/No) 7. Do you live together with someone who has been or is infected with COVID-19 (at the moment or before) (Yes/No) 8. Do you have risk factors for COVID-19? (Yes/No) 9. Have you been or are you infected with COVID-19 (at the moment or before) (Yes/No) 10. Are you worried that you might get infected with COVID-19? (On a Likert scale from 1 = not at all at all to 5 = very much). We assessed the perceived danger of Covid-19 for young and old people to control for solidarity with the own generation (since most of the participants and the individuals in the pictures were young) or the older generation, who were featured in the media and by doctors as more vulnerable and therefore as needing more protection.

Moreover, general anxiety was measured with the General Anxiety Disorder Scale (GAD-7^59^). Participants had to rate the severity of their symptoms, such as feeling nervous, anxious or on edge, trouble relaxing, becoming easily annoyed or irritable over the past two weeks. Response options were “not at all”, “several days”, “more than half the days” and “nearly every day”.

For control purposes we also collected ratings of the pictures on three dimensions: Arousal (On a Likert scale from 1 = not arousing to 5 = very arousing), Valence (On a Likert scale from 1 = negative to 5 = positive), and Danger (On a Likert scale from 1 = not dangerous at all to 5 = very dangerous).

At the end of the study, participants were thanked for their participation and debriefed.

### Ethics statement

The study was conducted in accordance with the guidelines laid down in the Declaration of Helsinki. There was no need to obtain further formal ethics review or approval as the methods were standard, there was no risk, participants gave their informed consent, and the procedure was already evaluated by professional psychologists to be consistent with ethical standards of the German Research Foundation (DFG). Moreover, the middle author (MHF) ensured that the study was carried out in accordance with the guidelines of the German Research Foundation (DFG), including written informed consent and confidentiality of data as well as personal conduct. All participants submitted their informed consent at the beginning of the experiment by clicking the relevant online link and were reimbursed with course credits for their participation.

### Picture copyright statement

We hereby declare that we had full permission to use the photos and did not violate any copyright. The pictures were downloaded from reputable platforms, therefore we assume that all persons have given their consent to be photographed and published to the respective publisher/licensor. The authors are not in breach of any publishing consent related with the identification of individuals in the photos.

## Analysis and preprocessing

Data of participants was first checked for quality. Seven participants were excluded because of a high error rate (above 20% in at least one experimental block). Further, two participants were excluded because they reported not having done the experiment seriously and ten participants were excluded because of technical issues, such as keyboard issues, poor internet connection or experiment lagging. The remaining datasets (n = 216) were further analyzed. Although this is about 6% lower than the estimated optimal sample size, we decided against further recruitment for reasons of efficiency. All trials with reaction times below 300 ms and above 1500 ms as well as trials outside of two standard deviations of the individual mean were excluded from the analysis. The remaining trials (n = 37,823) were further analyzed in a 2 mask (face-mask, no face-mask) × 3 distance (distance of 50, 90, 150 cm) × 2 interaction style (shaking hands, no interaction) repeated measures ANOVA. The factor gender of the individuals in the pictures (stimuli gender) was controlled for in another rm-ANOVA which was the same as the aforementioned but extended by factor gender (female-female, female-male, male–female, male-male). Other control variables (see 3.3.3 Measures) were included in separate rmANOVAs as covariates.

## Results

### Hypotheses testing

Rm-ANOVA of 2 (face-mask, no face-mask) × 3 (distance of 50, 90, 150 cm) × 2 (shaking hands, no interaction) was applied for all comparisons with more than two levels. The rm-ANOVA revealed a reliable main effect of face mask, *F*(1, 215) = 39.15, *η*^2^ = 0.15, *p* < 0.001, with a processing advantage for pictures in which a face mask was shown (mean RT = 628.35, *SD* = 70.50), over pictures no face mask (mean RT = 635.67, *SD* = 69.34). We were able to support H2. Further, we detected main effects of distance *F*(2, 422) = 49.11, *η*^2^ = 0.19, *p* < 0.001, with fastest RTs for 90 cm (mean RT = 624.75, *SD* = 70.09), followed by 50 cm (mean RT = 632.68, *SD* = 69.80), and 150 cm (mean RT = 638.60, *SD* = 69.86), and a small effect of interaction style, with *F*(1, 215) = 4.53, *η*^2^ = 0.02, *p* = 0.03, with faster RT for stimuli showing individuals without interaction (mean RT = 630.78, *SD* = 70.63) compared to pictures with interaction of shaking hands (mean RT = 633.23, *SD* = 69.20). Thus, H1 was partially confirmed and H3 was supported.

Moreover, there were reliable interaction effects of face mask * distance, *F*(2, 429) = 3.19, *η*^2^ = 0.02, *p* = 0.04, distance * interaction style, *F*(2, 424) = 36.10, *η*^2^ = 0.14, *p* < 0.001, and a triple interaction of face mask * distance * interaction style, *F*(2, 425) = 41.46, *η*^2^ = 0.16, *p* < 0.001. No statistical interaction of face mask * interaction style was found, *F*(1,215) = 0.06, *η*^2^ < 0.001, *p* = 0.81. The reported effect sizes are Eta-squared (not general or partial).

**Figure 2:**
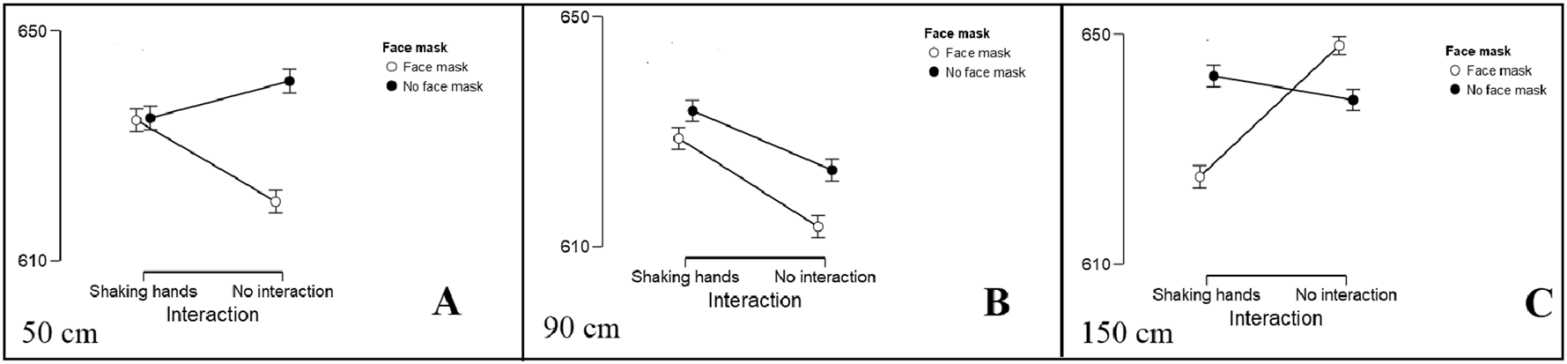
Reaction times [ms] as a function of face mask and interaction style in all three distances (50 cm/near, 90 cm/middle, 150 cm/far). Panel (**A**) 50 cm. Panel (**B**) 90 cm. Panel (**C**) 150 cm. Error bars denote one standard deviation of the mean.

In line with our expectations, the participants were fastest when reacting to pictures with a face mask and no interaction, at a distance of 90 cm (mean RT = 613.50 ms, *SD* = 71.56) and 50 cm (mean RT = 620.30, *SD* = 68.59). Slowest were the participants when reacting to the pictures with a face mask, with no interaction and at a distance of 150 cm (mean RT = 648 ms, *SD* = 70.92) and with no mask, with interaction and again at a distance of 150 cm (mean RT = 642.70, *SD* = 67.78). H4 was partially supported. See Fig. 2A–C for a visualization of these results. An overview of results is provided in Table 2. Descriptive statistics are shown in Supplementary Table 1. Post-hoc analyses (Holm-corrected) are summarized in Supplementary Table 2, Supplementary Table 3 and Supplementary Table 4. In particular, concerning only the main effect of distance, reaction times significantly differ between 50 and 90 cm, *t*(1.96) = 5.642, *SE* = 1.402, *p* < 0.001 and 150 cm *t*(1.96) = −4.235, *SE* = 1.402, *p* < 0.001, respectively, as well as 90 cm and 150 cm, *t*(1.96) = −9.877, *SE* = 1.402, *p* < 0.001. The fastest reaction time is at 90 cm with 624.75 ms. The participants were faster to react to pictures with face masks (*M* = 628.341) than with no face mask (*M* = 635.664), *t*(1.96) = −6.257, *SE* = 1.170, *p* < 0.001. They were also faster to react to pictures with no interaction (*M* = 630.785) than with shaking hands (*M* = 633.220), *t*(1.96) = 2.128, *SE* = 1.145, *p* < 0.05. However, for example, the interaction condition Mask/No interaction/90 cm is not significantly different from the condition Mask/No interaction/50 cm, *t*(1.98) = 2.546, *SE* = 2.678, *p* = 0.242. Further comparisons can be found in Supplementary Table 4.

**Table 2:** Rm-ANOVA of face-mask x distance x interaction style within subject effects.

	Sum of Squares	*df*	Mean Square	*F*	*p*
Face mask	34,755.22	1.000	34,755.22	39.150	< .001
Residual	190,863.02	215.000	887.73		
Distance	83,358.58	2.000	41,679.29	49.111	< .001
Residual	364,929.40	430.000	848.67		
Interaction style	3844.02	1.000	3844.02	4.528	0.034
Residual	182,523.19	215.000	848.95		
Face mask ✻ Distance	4747.26	2.000	2373.63	3.193	0.042
Residual	319,639.92	430.000	743.35		
Face mask ✻ Interaction style	42.23	1.000	42.23	0.058	0.811
Residual	157,575.60	215.000	732.91		
Distance ✻ Interaction style	53,603.00	2.000	26,801.50	36.098	< .001
Residual	319,264.26	430.000	742.48		
Face mask ✻ Distance ✻ Interaction style	63,452.87	2.000	31,726.44	41.456	< .001
Residual	329,081.50	430.000	765.31		

### Control variables

None of the statistical interactions with demographic control variables—participants’ gender, education or age—reached the level of significance (Supplementary Table 5, Supplementary Table 6 and Supplementary Table 7).

As for other control variables, we found several reliable statistical interactions: First, there was a reliable statistical interaction face mask * distance * interaction style * COVID-19 danger, *F*(2, 428) = 4.01, *p* = 0.02. This means that perceived danger of the virus modifies the relation of preferred behavior towards not interacting with others at near distances and thus the manner of social interaction.

Next, there was a reliable statistical interaction of face mask * interaction style * COVID-19 danger for the elderly, *F*(1, 214) = 4.57, *p* = 0.03. This finding means that perceived danger of the virus for older people and not for the participants themselves also modifies preferred manner of social interaction.

Next, there was a reliable statistical interaction of face mask * distance* interaction style * own COVID-19 infection, *F*(2, 428) = 3.34, *p* = 0.04). This finding reflects that having an infection also influenced attitude toward preferred social interaction: When having been infected, people tend not to interact with others.

None of the remaining control variables (danger for others, danger for the young people, mask as protection, living with a person with COVID-19 risk or living with a person who has been infected with COVID-19, own risk of infection, worry of COVID-19) reached statistical significance. The results of the individual rmANCOVAs are summarized in Supplementary Tables 8–17.

Results obtained with the General Anxiety Disorder scale (GAD-7) were analyzed as median split (med = 2, less anxious = 1 (< 2) und 2 = more anxious (> = 2)). None of the comparisons reached significance (see Supplementary Table 18). The effect of personal traits such as anxiety needs to be investigated further in future studies as it could explain—at least in part—some of the variance in the data^60^.

### Stimuli ratings

Further, we compared valence, arousal and danger ratings of separate stimuli collected at the end of the study as to the main factors: mask, interaction, distance. The variables were strongly correlated: arousal and valence *r*_*s*_ = −0.50, *p* < 0.001; arousal and danger *r*_*s*_ = 0.68, *p* < 0.001; valence and danger *r*_*s*_ = −0.57, *p* < 0.001. In other words, the less positive the picture was, the more arousal it evoked, and the more danger it suggested.

The ANOVA results are summarized in Supplementary Table 19, Supplementary Table 23 and Supplementary Table 29, respectively. Post-hoc comparison results (Holm-corrected) are summarized in Supplementary Table 20–22, Supplementary Tables 24–28, and Supplementary Tables 28–31, respectively.

Results show that, first, the participants evaluated more positively pictures with individuals wearing no face mask or standing at a close (50 cm and 90 cm) distance, or shaking hands (main effects of face mask, distance and interaction, respectively). Second, the participants perceived more arousal looking at pictures with a face mask in general, no face mask and no interaction or with face mask and shaking hands (main effect of mask and interaction effects of mask and interaction). Third, the participants evaluated pictures with a face mask or with individuals at a close distance (50 cm) as more dangerous.

Since the stimuli were rather heterogeneous and not validated before, we conducted linear mixed model analysis in R (Version 1.4.1106) using the lme4 package55^61^ to control for different intercepts for stimuli^61,62^. Prior to doing this, we removed trials with missing values (valence 1%, arousal 24%, and danger 3%). Missing values were only present in the ratings and were randomly distributed among participants.

We included reaction time as the dependent variable and added fixed effects of face mask, interaction, distance, their interaction effects, and the following categorical variables: living with someone with COVID-19, living with someone with a COVID-19 risk, own infection, own risk, gender; and the following continuous variables: valence, arousal, danger, age, mask as protection, danger for elderly, danger for young people, danger for other people, own danger, own fear of COVID-19, and GAD score. Continuous variables were centered; categorical variables were coded with sum contrasts, but for the distance that was coded with Successive Differences Contrast method. We included Participant and Stimuli as random effects.

The model with both random slopes and intercepts did not converge, so we removed the slope parameter from the model. We kept in the model only factors that reached the significance level of *p* < 0.1. The results of the model fit are reported in Supplementary Table 32.

Only the main effects of distance (contrast 150 cm–90 cm) reached the significance level (*b* = 14.650, *t* = 2.295, *p* < 0.05).

As seen from the table, fixed factors explained around 1% of the total variance of 58% explained by the model. To specify the proportion of variance for Participant and Stimuli respectively, we ran two separate linear models with reaction time as a dependent variable with Participant and Stimuli as factor, respectively, using the “stats” package in R (Version 1.4.1106). Participant as a random factor explained 52% of the total variance, and Stimuli as a random factor explained 4% of the total variance.

## Discussion

### Hypotheses testing

The aim of the study was to assess implicit evaluation of comfortable interpersonal space in the context of the COVID-19 pandemic. As an extension of previous studies on this subject, we also manipulated the factor of interaction style between individuals. We used gender classification speed as a proxy to measure participants’ approach and avoidance attitudes towards the observed social scenes.

The study yielded two major findings. First, as expected, participants were fastest when seeing individuals with a face mask, but not engaged in an interaction by shaking hands, slightly within and outside the peripersonal space (50–90 cm) as approaching the most appropriate distance for communication in Germany^63^. These results are in line with most of the previous studies^23,27,39,40^ and only contradict the very early ones^36,37^. This can indicate that human perception of distances and masks has changed since the early days of the pandemic: the mask is no longer a symbol of danger but rather of protection. This tendency might have taken place when masks were proclaimed obligatory^23^ and seems to be stable, as multiple studies have shown^40,41^. The effect of adapting to the social distancing norms by increasing the interpersonal distance was also shown to be stable and might continue into the post-pandemic phase^38^. Wearing a mask and keeping more distance has become normal and even a sign of respect. Moreover, face masks facilitate social behavior as perceived danger is diminished, reflected by faster reaction times.

In addition, face masks in this interpretation do not trigger avoidance behavior but rather reflect positive, less dangerous social interaction then when no face mask is shown. We interpret our results in light of a positivity bias: Shorter reaction times are known to reflect an approach tendency to positive stimuli while longer reaction times reflect avoiding tendencies^44^. Participants perceived a situation as more positive when both individuals are shown with face masks and when they do not interact with each other. In addition, the situation is perceived as being more positive when the stimuli show individuals with an interpersonal distance of 90 cm which is almost the recommended distance by World Health Organization of 100 cm. With a closer distance, the peripersonal spaces of the two individuals collide—there is not enough space in their comfort zone and thus no positivity bias emerges.

As mentioned earlier, though, another possible explanation of faster reaction times for distances of 50 and 90 cm can be the opposite: When seeing individuals at a close distance participants might want to escape this situation as fast as they can since it means danger (negativity bias^45^); this activation might lead to faster responding.

At the distance of 90 cm participants are slower when the depicted individuals shake hands, suggesting that the participants perceive this behavior as a risk.

Finally, at a closer distance of 50 cm, the difference in reaction times is only present in the no interaction condition: Reaction times are slower with no face mask. Participants react fastest to pictures of individuals wearing a face mask and not interacting.

At the distance of 150 cm the effects are opposite depending on the interaction style: When a picture depicts shaking hands, participants need longer to react when the individuals wear no face mask. In contrast, when a picture depicts no interaction, they react slower to individuals wearing a face mask. Probably, a face mask is considered unnecessary when individuals stand relatively far apart and do not interact. In general, such a large interpersonal distance might cause discomfort by violating cultural norms, which results in slower reaction times^26,56^.

To sum it up, when the individuals in the pictures were not wearing a face mask or were about to shake hands, the reaction times increased, suggesting that observers experienced a growing wish to avoid such situations. On the other hand, wearing a face mask can be an additional trigger adding salience to the COVID-19 danger and making them escape such a social situation.

The finding of longer reaction times at 150 cm can be due to a measuring artifact. When individuals in the pictures stood very far from each other (150 cm), it was more difficult for our participants to obtain a fast overview and recognize one of them as a man or a woman. Therefore, they needed more time to react. Another possible explanation is that communication at such a large distance is unusual for the European culture and produces a task difficulty effect^56^. A third explanation could go in line with Serino and colleagues^24^, revealing a stronger near-far differentiation, with less interaction at farther distances. This means that near-far differentiation is concentrated to a greater extent within the space around the body, while it is reduced at farther distances. Finally, slower reaction times can be merely an artifact which emerged due to the fact that assessing contents of a picture with individuals at longer interpersonal distances requires an ocular saccade and thus longer processing times in general, which is not the case with shorter distances.

On a whole, for the 50 and 90 cm distances, in line with our hypothesis, the further the individuals in the pictures were apart, the faster the reaction times were. The larger 150 cm, however, does not fit into this pattern.

Among the control factors, only a general perception of COVID-19 as danger and especially for the elderly people, as well as having been infected oneself, had an impact on the participants’ reactions. Apparently, these commonplace factors mentioned in the media as crucial to consider, as well as one’s own presumably unpleasant experience, influence implicit perception of interactional situations. Therefore these three factors—danger in general, danger for the elderly, and own experience—should be considered in promoting protective measures. However, the influence might have changed with the pandemic dynamics and general vaccination policies.

### Ratings

Surprisingly, contrary to the main analysis, explicit ratings showed a more positive evaluation of pictures with a face mask, at a closer distance or of individuals shaking hands.

These findings seem to contradict our interpretation of the implicit measures results in light of the positivity bias, where pictures with individuals wearing a face mask and with no interaction at the middle distance of 90 cm were reacted to faster. We construed this effect as dictated by a feeling of comfort and safety under these conditions. However, we cannot completely rule out the opposite theory of the negativity bias, when faster reaction times are the consequence of an avoidance mechanism. Since the results of the implicit and the explicit measures are not consistently opposite and the number of stimuli per condition evaluated was rather small (pictures were evaluated only once), a hybrid explanation can be suggested. It posits a discrepancy between the explicit and implicit measures of interpersonal distance and face mask perception. When explicitly asked, participants might be more cautious at evaluating such cues as a face mask or interaction, adding their beliefs of what a proper social interaction is, e. g., with or with no face mask. In this case a face mask signals danger as it did in the study by Marchiori and colleagues^36^. Implicitly, though, they might avoid larger interpersonal distances and situations with people wearing no face mask being perceived as dangerous. Further studies with an unequivocal aim of contrasting explicit and implicit reactions will shed more light on this dilemma.

A notable conclusion from the linear mixed modeling with the control variables is that only one of the distance contrasts reached the significance level. This fact might be accounted for by including random factors in the model and using long trial-wise data, with more noise in it, compared to the aggregated data for the rmANOVA.

## Conclusion

The present study was conducted within the framework of the COVID-19 pandemic. We tested how participants reacted to picture stimuli showing individuals with and without a face mask in social interaction or without social interaction within different distances. Adding an interaction as a factor was a valuable addition to previous studies.

### Social behavior during the pandemic

As expected, faster reaction times reflected less avoidance in a middle-wide distance over a near distance. We tend to avoid dangerous situations such as meeting people who are not wearing a face mask at close distances. Contrary to previous research^38^, the 90 cm distance seems to be more comfortable, though, than the recommended 150 cm one. Also, at the preferred communication distance we still do not want to socially interact. This means that touching others, as we do during a handshake, is nowadays a barrier that in times of the COVID-19 pandemic we rather do not want to cross. Thus, it is not only the face mask that changes social behavior and reduces social contacts. It is instead the reasoned fear of getting sick as a reaction to a combination of social cues such as a face mask and an interaction intention.

### Face masks help maintain social behavior

The findings imply that besides their protective effect, face masks reduce our interpersonal space and make us more willing to interact with other individuals. This finding is crucial because security equipment here does not seem to reflect danger but protection instead. Our results are consistent with most of the previous studies^23,39–41^ about face masks and interpersonal space. A face mask might reduce avoidance behavior by implying trustworthiness of the interaction partner^23^. As known from previous research, a relationship between trustworthiness and interpersonal space is twofold. On the one hand, when an interaction partner stands within one’s personal space (in our case, about 50 cm), their trustworthiness decreases. On the other hand, when an untrustworthy interaction partner intrudes one’s personal space, in its turn, it causes a withdrawal reaction. Interestingly, previous studies showed that face masks can impair attribution of trustworthiness and recognition of emotion, which, in its turn, can impact the interpersonal distance^64,65^. Apparently, in our study masks did not produce these negative effects.

It is worth mentioning that we did not find significant differences in reaction times between 50 and 90 cm, in cases when the persons in the picture did not interact and wore a face mask. Thus, both distances seem to be equally acceptable for the participants.

Thus, face masks are not only a tool to protect ourselves from infection; they also help us to maintain social behavior and healthy communication with each other, as far (or, rather, as close) as possible. This means that face-masks are nowadays becoming a social tool.

### Limitations and future studies

One limitation of the present study is that social behavior was not tested in a real life setting but with pictures of strangers. We cannot know how people would have behaved in real world settings. Unfortunately, this cannot be tested because during the pandemic, it would be unethical to test participants in the lab and after the pandemic, fear of infection will not be salient anymore.

Another limitation is that the depicted situation did not include the participants themselves. They were mere observers, which may have changed the perception to a more meta-cognitive level, rather than being involved personally. In the end, it was not their own interpersonal space that was studied, but an observed one. On the other hand, studies on mirror mechanisms show that shared brain areas are activated when humans see other individuals perform an action and when they perform this action themselves^66,67^. On these grounds, we do believe that the tendencies established in the study would also be applicable to the participants themselves as though they were the ones communicating.

However, the pictures we used were technically manipulated and might not depict real-world distances accurately. Thus, we should consider the three distances as relative and not absolute. Moreover, confounds like image quality or size perception could have influenced the participants’ response times. Moreover, a yet unpublished study of the authors with the same pictures as stimuli provides evidence that at 50 and 90 cm participants generally underestimated the IPD while at an IPD of 150 cm, participants overestimated the distance^68^.

One should bear in mind that the effects found in the present study are very small, which may jeopardize the generalizability of the findings. Therefore practical conclusions for everyday live should be derived from these effects with caution. However, the Gorilla platform was proven to deliver high precision in measuring reaction times, which permits a higher sensitivity for even small differences between conditions^69^. On-site studies in standardized lab conditions are needed to substantiate our results. On the other hand, we acknowledge that also some interactions were underpowered, which demands replications in larger samples online, since samples > 200 are hard to achieve in a laboratory setting.

Further, the actual boundary of peripersonal space was not assessed in our participants and can only be assumed from previous literature. This limitation can also be overcome in a lab study using a standard multisensory stimulation manipulation.

Last but not least, due to a discrepancy between the implicit and explicit measures, we cannot completely rule out an alternative explanation of the reaction time pattern in light of the negativity bias: Faster reaction times signal avoidance rather than positivity. A large scale study with validated stimuli varying in valence could be diagnostic for this purpose.

Before the COVID-19 pandemic, wearing a face mask had not been relevant for most of us. Results of our study point out that novel situated cues, such as face masks and infection risk, impact our proximity assessment. Future studies could indeed test long-term effects of our experience with face masks in the last months. Preferences towards wearing a face mask, towards physical distance and towards willingness to interact with each other in the context of wearing face masks can be assessed after the pandemic as well. In addition, effects of factors such as anxiety should be investigated in further detail in the framework of social distancing and protection means to assess to which extent they influence perception of danger and avoidance behavior. We plan to examine whether pandemic-related changes in habits have left long-term traces in human spatial behavior and also whether pandemic-related habits are stronger determinants of behavior than inter-individual differences that exist independently of the pandemic.

## Supplementary Information

Supplementary Information 1.

Supplementary Information 2.
