# Supplementary material for: During the COVID-19 pandemic participants prefer settings with a face mask, no interaction and at a closer distance
Source: Sci Rep. 2022 Jul 27;12:12777. doi: 10.1038/s41598-022-16730-1 (PMC9326138; doi:10.1038/s41598-022-16730-1)
Supplement: Supplementary file 1 — Supplementary Information 1. [file 41598_2022_16730_MOESM1_ESM.pdf]

Exact Simulation Results

Shiny App developed by Daniël Lakens and Aaron Caldwell

April 19, 2022

Below are the results from a power analysis using an exact simulation of a factorial design wherein the data are sampled from an empircal distribution. If you encounter any problems please visit our GitHub page (https://github.com/arcaldwell49/Superpower) to the raise the issue.

Study Design

Model Formula

y ~ a * b * c + Error(subject/a * b * c)

The sample size was **19** *per cell.*

Correlation Matrix

	a1_b1_c1	a1_b1_c2	a1_b1_c3	a1_b2_c1	a1_b2_c2	a1_b2_c3	a2_b1_c1	a2_b1_c2	a2_b1_c3	a2_b2_c1	a2_b2_c2	a2_b2_c3
a1_b1_c1	1.0	0.9	0.9	0.9	0.9	0.9	0.9	0.9	0.9	0.9	0.9	0.9
a1_b1_c2	0.9	1.0	0.9	0.9	0.9	0.9	0.9	0.9	0.9	0.9	0.9	0.9
a1_b1_c3	0.9	0.9	1.0	0.9	0.9	0.9	0.9	0.9	0.9	0.9	0.9	0.9
a1_b2_c1	0.9	0.9	0.9	1.0	0.9	0.9	0.9	0.9	0.9	0.9	0.9	0.9
a1_b2_c2	0.9	0.9	0.9	0.9	1.0	0.9	0.9	0.9	0.9	0.9	0.9	0.9
a1_b2_c3	0.9	0.9	0.9	0.9	0.9	1.0	0.9	0.9	0.9	0.9	0.9	0.9
a2_b1_c1	0.9	0.9	0.9	0.9	0.9	0.9	1.0	0.9	0.9	0.9	0.9	0.9
a2_b1_c2	0.9	0.9	0.9	0.9	0.9	0.9	0.9	1.0	0.9	0.9	0.9	0.9
a2_b1_c3	0.9	0.9	0.9	0.9	0.9	0.9	0.9	0.9	1.0	0.9	0.9	0.9
a2_b2_c1	0.9	0.9	0.9	0.9	0.9	0.9	0.9	0.9	0.9	1.0	0.9	0.9
a2_b2_c2	0.9	0.9	0.9	0.9	0.9	0.9	0.9	0.9	0.9	0.9	1.0	0.9
a2_b2_c3	0.9	0.9	0.9	0.9	0.9	0.9	0.9	0.9	0.9	0.9	0.9	1.0

Variance-Covariance Matrix

	a1_b1_c1	a1_b1_c2	a1_b1_c3	a1_b2_c1	a1_b2_c2	a1_b2_c3	a2_b1_c1	a2_b1_c2	a2_b1_c3	a2_b2_c1	a2_b2_c2	a2_b2_c3
a1_b1_c1	10000	9000	9000	9000	9000	9000	9000	9000	9000	9000	9000	9000
a1_b1_c2	9000	10000	9000	9000	9000	9000	9000	9000	9000	9000	9000	9000
a1_b1_c3	9000	9000	10000	9000	9000	9000	9000	9000	9000	9000	9000	9000
a1_b2_c1	9000	9000	9000	10000	9000	9000	9000	9000	9000	9000	9000	9000
a1_b2_c2	9000	9000	9000	9000	10000	9000	9000	9000	9000	9000	9000	9000
a1_b2_c3	9000	9000	9000	9000	9000	10000	9000	9000	9000	9000	9000	9000
a2_b1_c1	9000	9000	9000	9000	9000	9000	10000	9000	9000	9000	9000	9000
a2_b1_c2	9000	9000	9000	9000	9000	9000	9000	10000	9000	9000	9000	9000
a2_b1_c3	9000	9000	9000	9000	9000	9000	9000	9000	10000	9000	9000	9000
a2_b2_c1	9000	9000	9000	9000	9000	9000	9000	9000	9000	10000	9000	9000
a2_b2_c2	9000	9000	9000	9000	9000	9000	9000	9000	9000	9000	10000	9000
a2_b2_c3	9000	9000	9000	9000	9000	9000	9000	9000	9000	9000	9000	10000

Plot of the design with mean and standard deviation.

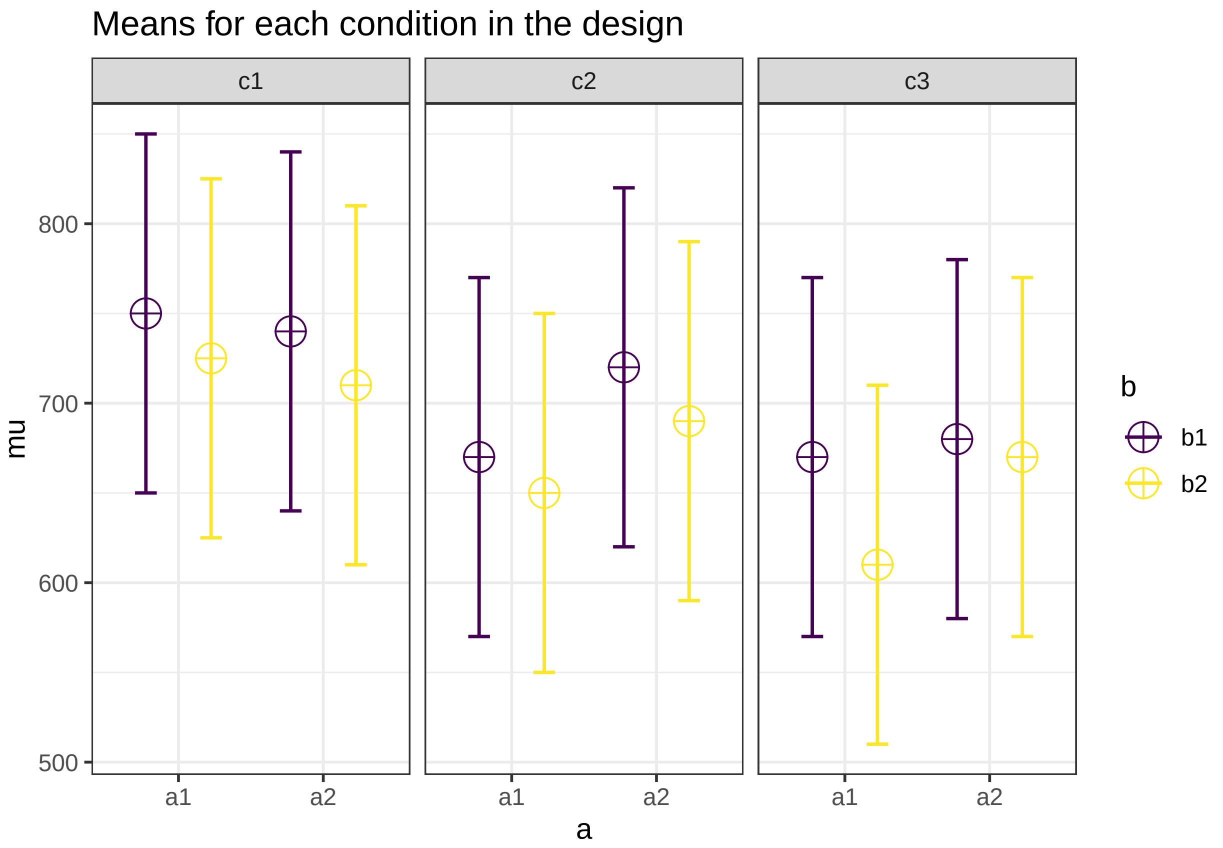

Legend: a = Mask (1 = mask, 2 = no mask; b = Interaction (1 = interaction, 2 = no interaction; c = Distance (1 = 50 cm, 2 = 90 cm, 3 = 150 cm)

Power for this specific design, with an alpha of 0.05, is included below.

ANOVA Power (%) and Effect Sizes (Partial Eta Squared)

	power	partial_eta_squared	cohen_f	non_centrality
a	99.86853	0.6063210	1.2410241	27.7225352
b	99.99961	0.7212954	1.6087350	46.5845070
c	100.00000	0.8508873	2.3887943	205.4281690
a:b	25.29066	0.0938098	0.3217470	1.8633803
a:c	99.95260	0.4890248	0.9782854	34.4535211
b:c	12.47727	0.0267306	0.1657250	0.9887324
a:b:c	78.48087	0.2193513	0.5300810	10.1154930

Legend: a = Mask (1 = mask, 2 = no mask; b = Interaction (1 = interaction, 2 = no interaction; c = Distance (1 = 50 cm, 2 = 90 cm, 3 = 150 cm)

Estimated Marginal Means Comparisons Power (%) and Effect Sizes (Cohen's f)

If estimated marginal means were selected, the results are included below.

contrast	power	partial_eta_squared	cohen_f	non_centrality
a_a1 b_b1 c_c1 - a_a2 b_b1 c_c1	15.564077	0.0083799	0.0919277	0.8989261
a_a1 b_b1 c_c1 - a_a1 b_b2 c_c1	64.557782	0.0501672	0.2298193	5.5457000
a_a1 b_b1 c_c1 - a_a2 b_b2 c_c1	95.775308	0.1191067	0.3677108	13.8404541
a_a1 b_b1 c_c1 - a_a1 b_b1 c_c2	99.999999	0.2885772	0.6368939	57.5699332
a_a1 b_b1 c_c1 - a_a2 b_b1 c_c2	81.755744	0.0539640	0.2388352	8.3237722
a_a1 b_b1 c_c1 - a_a1 b_b2 c_c2	100.000000	0.3879310	0.7961173	88.9853664
a_a1 b_b1 c_c1 - a_a2 b_b2 c_c2	99.993151	0.1857798	0.4776704	33.7744235
a_a1 b_b1 c_c1 - a_a1 b_b1 c_c3	99.999999	0.2885772	0.6368939	57.5699332
a_a1 b_b1 c_c1 - a_a2 b_b1 c_c3	99.999886	0.2369694	0.5572821	45.3183151
a_a1 b_b1 c_c1 - a_a1 b_b2 c_c3	100.000000	0.5540201	1.1145643	174.4113181
a_a1 b_b1 c_c1 - a_a2 b_b2 c_c3	100.000000	0.2885772	0.6368939	60.0434195
a_a2 b_b1 c_c1 - a_a1 b_b2 c_c1	28.206928	0.0186593	0.1378916	1.9463139
a_a2 b_b1 c_c1 - a_a2 b_b2 c_c1	79.955160	0.0706806	0.2757831	7.9858080
a_a2 b_b1 c_c1 - a_a1 b_b1 c_c2	99.999886	0.2369694	0.5572821	45.3183151
a_a2 b_b1 c_c1 - a_a2 b_b1 c_c2	46.979638	0.0247253	0.1592235	3.5981208
a_a2 b_b1 c_c1 - a_a1 b_b2 c_c2	100.000000	0.3392275	0.7165056	75.9924528
a_a2 b_b1 c_c1 - a_a2 b_b2 c_c2	99.677665	0.1367781	0.3980587	22.2463416
a_a2 b_b1 c_c1 - a_a1 b_b1 c_c3	99.999886	0.2369694	0.5572821	45.3183151
a_a2 b_b1 c_c1 - a_a2 b_b1 c_c3	99.988866	0.1857798	0.4776704	32.3830874
a_a2 b_b1 c_c1 - a_a1 b_b2 c_c3	100.000000	0.5171710	1.0349525	158.5521546
a_a2 b_b1 c_c1 - a_a2 b_b2 c_c3	99.999786	0.2369694	0.5572821	43.6028295
a_a1 b_b2 c_c1 - a_a2 b_b2 c_c1	29.132483	0.0186593	0.1378916	2.0225838
a_a1 b_b2 c_c1 - a_a1 b_b1 c_c2	99.929483	0.1608805	0.4378645	26.9180733
a_a1 b_b2 c_c1 - a_a2 b_b1 c_c2	7.691753	0.0015820	0.0398059	0.2345446
a_a1 b_b2 c_c1 - a_a1 b_b2 c_c2	99.999983	0.2628164	0.5970880	50.5985741
a_a1 b_b2 c_c1 - a_a2 b_b2 c_c2	91.678189	0.0720471	0.2786411	11.3295788
a_a1 b_b2 c_c1 - a_a1 b_b1 c_c3	99.929483	0.1608805	0.4378645	26.9180733
a_a1 b_b2 c_c1 - a_a2 b_b1 c_c3	99.111257	0.1137463	0.3582528	18.9981132
a_a1 b_b2 c_c1 - a_a1 b_b2 c_c3	100.000000	0.4559908	0.9155349	118.9628698
a_a1 b_b2 c_c1 - a_a2 b_b2 c_c3	99.950664	0.1608805	0.4378645	27.9771231
a_a2 b_b2 c_c1 - a_a1 b_b1 c_c2	97.056508	0.0920716	0.3184469	15.0108549
a_a2 b_b2 c_c1 - a_a2 b_b1 c_c2	15.501844	0.0062981	0.0796117	0.8898537
a_a2 b_b2 c_c1 - a_a1 b_b2 c_c2	99.991900	0.1857798	0.4776704	33.2950887
a_a2 b_b2 c_c1 - a_a2 b_b2 c_c2	46.979638	0.0247253	0.1592235	3.5981208
a_a2 b_b2 c_c1 - a_a1 b_b1 c_c3	97.056508	0.0920716	0.3184469	15.0108549

contrast	power	partial_eta_squared	cohen_f	non_centrality
a_a2 b_b2 c_c1 - a_a2 b_b1 c_c3	80.250225	0.0539640	0.2388352	8.0086830
a_a2 b_b2 c_c1 - a_a1 b_b2 c_c3	100.000000	0.3879310	0.7961173	92.4863574
a_a2 b_b2 c_c1 - a_a2 b_b2 c_c3	96.469559	0.0920716	0.3184469	14.3924833
a_a1 b_b1 c_c2 - a_a2 b_b1 c_c2	99.690392	0.1744186	0.4596386	22.4731536
a_a1 b_b1 c_c2 - a_a1 b_b2 c_c2	46.291348	0.0326975	0.1838554	3.5492480
a_a1 b_b1 c_c2 - a_a2 b_b2 c_c2	45.338819	0.0326975	0.1838554	3.4601135
a_a1 b_b1 c_c2 - a_a1 b_b1 c_c3	5.000000	0.0000000	0.0000000	0.0000000
a_a1 b_b1 c_c2 - a_a2 b_b1 c_c3	15.932481	0.0062981	0.0796117	0.9248636
a_a1 b_b1 c_c2 - a_a1 b_b2 c_c3	99.987433	0.1857798	0.4776704	32.0347319
a_a1 b_b1 c_c2 - a_a2 b_b2 c_c3	5.000000	0.0000000	0.0000000	0.0000000
a_a2 b_b1 c_c2 - a_a1 b_b2 c_c2	99.999641	0.2928287	0.6434940	42.3863908
a_a2 b_b1 c_c2 - a_a2 b_b2 c_c2	79.955160	0.0706806	0.2757831	7.9858080
a_a2 b_b1 c_c2 - a_a1 b_b1 c_c3	99.757373	0.1367781	0.3980587	23.1215893
a_a2 b_b1 c_c2 - a_a2 b_b1 c_c3	96.469559	0.0920716	0.3184469	14.3924833
a_a2 b_b1 c_c2 - a_a1 b_b2 c_c3	100.000000	0.4340375	0.8757291	113.5195900
a_a2 b_b1 c_c2 - a_a2 b_b2 c_c3	99.677665	0.1367781	0.3980587	22.2463416
a_a1 b_b2 c_c2 - a_a2 b_b2 c_c2	96.391532	0.1191067	0.3677108	14.3828183
a_a1 b_b2 c_c2 - a_a1 b_b1 c_c3	46.570352	0.0247253	0.1592235	3.5594147
a_a1 b_b2 c_c2 - a_a2 b_b1 c_c3	82.301715	0.0539640	0.2388352	8.4436059
a_a1 b_b2 c_c2 - a_a1 b_b2 c_c3	96.469559	0.0920716	0.3184469	14.3924833
a_a1 b_b2 c_c2 - a_a2 b_b2 c_c3	48.042140	0.0247253	0.1592235	3.6994543
a_a2 b_b2 c_c2 - a_a1 b_b1 c_c3	48.595294	0.0247253	0.1592235	3.7527137
a_a2 b_b2 c_c2 - a_a2 b_b1 c_c3	15.501844	0.0062981	0.0796117	0.8898537
a_a2 b_b2 c_c2 - a_a1 b_b2 c_c3	99.999999	0.2885772	0.6368939	59.1912687
a_a2 b_b2 c_c2 - a_a2 b_b2 c_c3	46.979638	0.0247253	0.1592235	3.5981208
a_a1 b_b1 c_c3 - a_a2 b_b1 c_c3	15.564077	0.0083799	0.0919277	0.8989261
a_a1 b_b1 c_c3 - a_a1 b_b2 c_c3	99.986344	0.2332613	0.5515663	31.9432321
a_a1 b_b1 c_c3 - a_a2 b_b2 c_c3	5.000000	0.0000000	0.0000000	0.0000000
a_a2 b_b1 c_c3 - a_a1 b_b2 c_c3	99.999641	0.2928287	0.6434940	42.3863908
a_a2 b_b1 c_c3 - a_a2 b_b2 c_c3	15.421305	0.0083799	0.0919277	0.8873120
a_a1 b_b2 c_c3 - a_a2 b_b2 c_c3	99.988189	0.2332613	0.5515663	32.3613412

Multiple Comparisons, *t*-test, Power (%) and Effect Sizes (Cohen's d_z)

Below are the power analysis results if all pairwise comparisons are completed via *t*-tests

	power	effect_size
p_a_a1_b_b1_c_c1_a_a1_b_b1_c_c2	99.999996	-1.7888544
p_a_a1_b_b1_c_c1_a_a1_b_b1_c_c3	99.999996	-1.7888544
p_a_a1_b_b1_c_c1_a_a1_b_b2_c_c1	63.511685	-0.5590170
p_a_a1_b_b1_c_c1_a_a1_b_b2_c_c2	100.000000	-2.2360680
p_a_a1_b_b1_c_c1_a_a1_b_b2_c_c3	100.000000	-3.1304952
p_a_a1_b_b1_c_c1_a_a2_b_b1_c_c1	15.193814	-0.2236068
p_a_a1_b_b1_c_c1_a_a2_b_b1_c_c2	78.964529	-0.6708204
p_a_a1_b_b1_c_c1_a_a2_b_b1_c_c3	99.999606	-1.5652476
p_a_a1_b_b1_c_c1_a_a2_b_b2_c_c1	95.759705	-0.8944272
p_a_a1_b_b1_c_c1_a_a2_b_b2_c_c2	99.981098	-1.3416408
p_a_a1_b_b1_c_c1_a_a2_b_b2_c_c3	99.999996	-1.7888544
p_a_a1_b_b1_c_c2_a_a1_b_b1_c_c3	5.000000	0.0000000
p_a_a1_b_b1_c_c2_a_a1_b_b2_c_c1	99.902487	1.2298374
p_a_a1_b_b1_c_c2_a_a1_b_b2_c_c2	45.437226	-0.4472136
p_a_a1_b_b1_c_c2_a_a1_b_b2_c_c3	99.981098	-1.3416408
p_a_a1_b_b1_c_c2_a_a2_b_b1_c_c1	99.999606	1.5652476
p_a_a1_b_b1_c_c2_a_a2_b_b1_c_c2	99.585515	1.1180340
p_a_a1_b_b1_c_c2_a_a2_b_b1_c_c3	15.193814	0.2236068
p_a_a1_b_b1_c_c2_a_a2_b_b2_c_c1	95.759705	0.8944272
p_a_a1_b_b1_c_c2_a_a2_b_b2_c_c2	45.437226	0.4472136
p_a_a1_b_b1_c_c2_a_a2_b_b2_c_c3	5.000000	0.0000000
p_a_a1_b_b1_c_c3_a_a1_b_b2_c_c1	99.902487	1.2298374
p_a_a1_b_b1_c_c3_a_a1_b_b2_c_c2	45.437226	-0.4472136
p_a_a1_b_b1_c_c3_a_a1_b_b2_c_c3	99.981098	-1.3416408
p_a_a1_b_b1_c_c3_a_a2_b_b1_c_c1	99.999606	1.5652476
p_a_a1_b_b1_c_c3_a_a2_b_b1_c_c2	99.585515	1.1180340
p_a_a1_b_b1_c_c3_a_a2_b_b1_c_c3	15.193814	0.2236068
p_a_a1_b_b1_c_c3_a_a2_b_b2_c_c1	95.759705	0.8944272
p_a_a1_b_b1_c_c3_a_a2_b_b2_c_c2	45.437226	0.4472136
p_a_a1_b_b1_c_c3_a_a2_b_b2_c_c3	5.000000	0.0000000
p_a_a1_b_b2_c_c1_a_a1_b_b2_c_c2	99.999958	-1.6770510
p_a_a1_b_b2_c_c1_a_a1_b_b2_c_c3	100.000000	-2.5714782
p_a_a1_b_b2_c_c1_a_a2_b_b1_c_c1	28.290457	0.3354102
p_a_a1_b_b2_c_c1_a_a2_b_b1_c_c2	7.477447	-0.1118034
p_a_a1_b_b2_c_c1_a_a2_b_b1_c_c3	98.544363	-1.0062306
p_a_a1_b_b2_c_c1_a_a2_b_b2_c_c1	28.290457	-0.3354102
p_a_a1_b_b2_c_c1_a_a2_b_b2_c_c2	89.698108	-0.7826238
p_a_a1_b_b2_c_c1_a_a2_b_b2_c_c3	99.902487	-1.2298374
p_a_a1_b_b2_c_c2_a_a1_b_b2_c_c3	95.759705	-0.8944272
p_a_a1_b_b2_c_c2_a_a2_b_b1_c_c1	100.000000	2.0124612
p_a_a1_b_b2_c_c2_a_a2_b_b1_c_c2	99.999606	1.5652476
p_a_a1_b_b2_c_c2_a_a2_b_b1_c_c3	78.964529	0.6708204
p_a_a1_b_b2_c_c2_a_a2_b_b2_c_c1	99.981098	1.3416408
p_a_a1_b_b2_c_c2_a_a2_b_b2_c_c2	95.759705	0.8944272
p_a_a1_b_b2_c_c2_a_a2_b_b2_c_c3	45.437226	0.4472136
p_a_a1_b_b2_c_c3_a_a2_b_b1_c_c1	100.000000	2.9068884
p_a_a1_b_b2_c_c3_a_a2_b_b1_c_c2	100.000000	2.4596748
p_a_a1_b_b2_c_c3_a_a2_b_b1_c_c3	99.999606	1.5652476
p_a_a1_b_b2_c_c3_a_a2_b_b2_c_c1	100.000000	2.2360680

	power	effect_size
p_a_a1_b_b2_c_c3_a_a2_b_b2_c_c2	99.999996	1.7888544
p_a_a1_b_b2_c_c3_a_a2_b_b2_c_c3	99.981098	1.3416408
p_a_a2_b_b1_c_c1_a_a2_b_b1_c_c2	45.437226	-0.4472136
p_a_a2_b_b1_c_c1_a_a2_b_b1_c_c3	99.981098	-1.3416408
p_a_a2_b_b1_c_c1_a_a2_b_b2_c_c1	78.964529	-0.6708204
p_a_a2_b_b1_c_c1_a_a2_b_b2_c_c2	99.585515	-1.1180340
p_a_a2_b_b1_c_c1_a_a2_b_b2_c_c3	99.999606	-1.5652476
p_a_a2_b_b1_c_c2_a_a2_b_b1_c_c3	95.759705	-0.8944272
p_a_a2_b_b1_c_c2_a_a2_b_b2_c_c1	15.193814	-0.2236068
p_a_a2_b_b1_c_c2_a_a2_b_b2_c_c2	78.964529	-0.6708204
p_a_a2_b_b1_c_c2_a_a2_b_b2_c_c3	99.585515	-1.1180340
p_a_a2_b_b1_c_c3_a_a2_b_b2_c_c1	78.964529	0.6708204
p_a_a2_b_b1_c_c3_a_a2_b_b2_c_c2	15.193814	0.2236068
p_a_a2_b_b1_c_c3_a_a2_b_b2_c_c3	15.193814	-0.2236068
p_a_a2_b_b2_c_c1_a_a2_b_b2_c_c2	45.437226	-0.4472136
p_a_a2_b_b2_c_c1_a_a2_b_b2_c_c3	95.759705	-0.8944272
p_a_a2_b_b2_c_c2_a_a2_b_b2_c_c3	45.437226	-0.4472136
