# Supplementary material for: During the COVID-19 pandemic participants prefer settings with a face mask, no interaction and at a closer distance
Source: Sci Rep. 2022 Jul 27;12:12777. doi: 10.1038/s41598-022-16730-1 (PMC9326138; doi:10.1038/s41598-022-16730-1)
Supplement: Supplementary file 2 — Supplementary Information 2. [file 41598_2022_16730_MOESM2_ESM.pdf]

Supplementary Table 1

Descriptive statistics

Face mask	Distance	Interaction	Mean	SD
Face mask	50 cm	Shaking hands	634.384	71.885
		No interaction	620.282	68.589
	90 cm	Shaking hands	628.747	69.845
		No interaction	613.463	71.565
	150 cm	Shaking hands	625.161	70.179
		No interaction	648.007	70.916
No face mask	50 cm	Shaking hands	634.772	67.641
		No interaction	641.201	71.082
	90 cm	Shaking hands	633.564	67.895
		No interaction	623.233	71.056
	150 cm	Shaking hands	642.693	67.784
		No interaction	638.523	70.569

Supplementary Table 2

Post-hoc analysis with factors mask and distance

		Mean Difference	SE	t	p_{holm}
50 cm, Face mask	90 cm, Face mask	6.228	1.920	3.244	0.006
	150 cm, Face mask	-9.251	1.920	-4.819	< .001
	50 cm, No face mask	-10.654	1.914	-5.565	< .001
	90 cm, No face mask	-1.065	1.977	-0.539	0.956
	150 cm, No face mask	-13.275	1.977	-6.715	< .001
90 cm, Face mask	150 cm, Face mask	-15.479	1.920	-8.063	< .001
	50 cm, No face mask	-16.882	1.977	-8.540	< .001
	90 cm, No face mask	-7.293	1.914	-3.810	< .001
	150 cm, No face mask	-19.503	1.977	-9.865	< .001
150 cm, Face mask	50 cm, No face mask	-1.403	1.977	-0.709	0.956
	90 cm, No face mask	8.186	1.977	4.141	< .001
	150 cm, No face mask	-4.024	1.914	-2.102	0.144
50 cm, No face mask	90 cm, No face mask	9.588	1.920	4.995	< .001
	150 cm, No face mask	-2.621	1.920	-1.365	0.517
90 cm, No face mask	150 cm, No face mask	-12.209	1.920	-6.360	< .001

Note. P-value adjusted for comparing a family of 15
Note. Results are averaged over the levels of: Interaction

Supplementary Table 3

Post-hoc analysis with factors interaction and distance

		Mean Difference	SE	t	p_{holm}
50 cm, Shaking hands	90 cm, Shaking hands	3.423	1.919	1.783	0.374
	150 cm, Shaking hands	0.651	1.919	0.339	1.000
	50 cm, No interaction	3.837	1.898	2.022	0.262
	90 cm, No interaction	16.230	1.962	8.274	< .001
	150 cm, No interaction	-8.687	1.962	-4.429	< .001
90 cm, Shaking hands	150 cm, Shaking hands	-2.771	1.919	-1.444	0.447
	50 cm, No interaction	0.414	1.962	0.211	1.000
	90 cm, No interaction	12.808	1.898	6.749	< .001
	150 cm, No interaction	-12.109	1.962	-6.173	< .001
150 cm, Shaking hands	50 cm, No interaction	3.186	1.962	1.624	0.419
	90 cm, No interaction	15.579	1.962	7.942	< .001
	150 cm, No interaction	-9.338	1.898	-4.920	< .001
50 cm, No interaction	90 cm, No interaction	12.394	1.919	6.458	< .001
	150 cm, No interaction	-12.524	1.919	-6.525	< .001
90 cm, No interaction	150 cm, No interaction	-24.917	1.919	-12.983	< .001

Note. P-value adjusted for comparing a family of 15

Note. Results are averaged over the levels of: Face mask

Supplementary Table 4

Post-hoc analysis with factors mask, interaction and distance

		Mean Difference	SE	t	p_{holm}
50 cm, Shaking hands, Face mask	90 cm, Shaking hands, Face mask	5.637	2.679	2.104	0.604
	150 cm, Shaking hands, Face mask	9.223	2.679	3.443	0.017
	50 cm, No interaction, Face mask	14.102	2.664	5.294	< .001
	90 cm, No interaction, Face mask	20.921	2.700	7.749	< .001
	150 cm, No interaction, Face mask	-13.623	2.700	-5.046	< .001
	50 cm, Shaking hands, No face mask	-0.389	2.675	-0.145	1.000
	90 cm, Shaking hands, No face mask	0.820	2.711	0.303	1.000
	150 cm, Shaking hands, No face mask	-8.309	2.711	-3.065	0.055
	50 cm, No interaction, No face mask	-6.817	2.696	-2.529	0.243
	90 cm, No interaction, No face mask	11.151	2.750	4.055	0.002
	150 cm, No interaction, No face mask	-4.139	2.750	-1.505	1.000
90 cm, Shaking hands, Face mask	150 cm, Shaking hands, Face mask	3.586	2.679	1.339	1.000
	50 cm, No interaction, Face mask	8.465	2.700	3.135	0.047
	90 cm, No interaction, Face mask	15.285	2.664	5.738	< .001
	150 cm, No interaction, Face mask	-19.260	2.700	-7.133	< .001
	50 cm, Shaking hands, No face mask	-6.025	2.711	-2.223	0.475
	90 cm, Shaking hands, No face mask	-4.817	2.675	-1.800	0.930
	150 cm, Shaking hands, No face mask	-13.945	2.711	-5.144	< .001
	50 cm, No interaction, No face mask	-12.453	2.750	-4.528	< .001
	90 cm, No interaction, No face mask	5.514	2.696	2.046	0.656
	150 cm, No interaction, No face mask	-9.776	2.750	-3.555	0.012
150 cm, Shaking hands,	50 cm, No interaction, Face	4.879	2.700	1.807	0.930

		Mean Difference	SE	t	p_{holm}
Face mask	mask				
	90 cm, No interaction, Face mask	11.699	2.700	4.333	< .001
	150 cm, No interaction, Face mask	-22.845	2.664	-8.577	< .001
	50 cm, Shaking hands, No face mask	-9.611	2.711	-3.546	0.012
	90 cm, Shaking hands, No face mask	-8.403	2.711	-3.100	0.051
	150 cm, Shaking hands, No face mask	-17.531	2.675	-6.553	< .001
	50 cm, No interaction, No face mask	-16.039	2.750	-5.832	< .001
	90 cm, No interaction, No face mask	1.929	2.750	0.701	1.000
	150 cm, No interaction, No face mask	-13.362	2.696	-4.957	< .001
50 cm, No interaction, Face mask	90 cm, No interaction, Face mask	6.819	2.679	2.546	0.242
	150 cm, No interaction, Face mask	-27.725	2.679	-10.350	< .001
	50 cm, Shaking hands, No face mask	-14.490	2.696	-5.376	< .001
	90 cm, Shaking hands, No face mask	-13.282	2.750	-4.830	< .001
	150 cm, Shaking hands, No face mask	-22.411	2.750	-8.149	< .001
	50 cm, No interaction, No face mask	-20.919	2.675	-7.819	< .001
	90 cm, No interaction, No face mask	-2.951	2.711	-1.089	1.000
	150 cm, No interaction, No face mask	-18.241	2.711	-6.729	< .001
90 cm, No interaction, Face mask	150 cm, No interaction, Face mask	-34.544	2.679	-12.896	< .001
	50 cm, Shaking hands, No face mask	-21.310	2.750	-7.749	< .001
	90 cm, Shaking hands, No face mask	-20.101	2.696	-7.457	< .001
	150 cm, Shaking hands, No face mask	-29.230	2.750	-10.629	< .001
	50 cm, No interaction, No face mask	-27.738	2.711	-10.232	< .001
	90 cm, No interaction, No face mask	-9.770	2.675	-3.652	0.009
	150 cm, No interaction, No face mask	-25.060	2.711	-9.245	< .001

		Mean Difference	SE	t	p_{holm}
150 cm, No interaction, Face mask	50 cm, Shaking hands, No face mask	13.234	2.750	4.812	< .001
	90 cm, Shaking hands, No face mask	14.443	2.750	5.252	< .001
	150 cm, Shaking hands, No face mask	5.314	2.696	1.971	0.733
	50 cm, No interaction, No face mask	6.806	2.711	2.511	0.243
	90 cm, No interaction, No face mask	24.774	2.711	9.139	< .001
	150 cm, No interaction, No face mask	9.484	2.675	3.545	0.012
50 cm, Shaking hands, No face mask	90 cm, Shaking hands, No face mask	1.209	2.679	0.451	1.000
	150 cm, Shaking hands, No face mask	-7.920	2.679	-2.957	0.076
	50 cm, No interaction, No face mask	-6.428	2.664	-2.413	0.303
	90 cm, No interaction, No face mask	11.540	2.700	4.274	< .001
	150 cm, No interaction, No face mask	-3.750	2.700	-1.389	1.000
90 cm, Shaking hands, No face mask	150 cm, Shaking hands, No face mask	-9.129	2.679	-3.408	0.019
	50 cm, No interaction, No face mask	-7.637	2.700	-2.828	0.109
	90 cm, No interaction, No face mask	10.331	2.664	3.879	0.004
	150 cm, No interaction, No face mask	-4.959	2.700	-1.837	0.930
150 cm, Shaking hands, No face mask	50 cm, No interaction, No face mask	1.492	2.700	0.553	1.000
	90 cm, No interaction, No face mask	19.460	2.700	7.207	< .001
	150 cm, No interaction, No face mask	4.170	2.664	1.565	1.000
50 cm, No interaction, No face mask	90 cm, No interaction, No face mask	17.968	2.679	6.708	< .001
	150 cm, No interaction, No face mask	2.678	2.679	1.000	1.000
90 cm, No interaction, No face mask	150 cm, No interaction, No face mask	-15.290	2.679	-5.708	< .001

Note. P-value adjusted for comparing a family of 66

Supplementary Table 5

Results of the rmANOVA with the factor gender

	Sum of Squares	df	Mean Square	F	p
Face mask	14404.900	1.000	14404.900	16.111	< .001
Face mask ✻ Gender	837.206	1.000	837.206	0.936	0.334
Residual	188653.448	211.000	894.092		
Distance	39158.791	2.000	19579.396	23.118	< .001
Distance ✻ Gender	3192.854	2.000	1596.427	1.885	0.153
Residual	357403.446	422.000	846.928		
Interaction	2288.123	1.000	2288.123	2.671	0.104
Interaction ✻ Gender	5.281	1.000	5.281	0.006	0.937
Residual	180754.148	211.000	856.655		
Face mask ✻ Distance	2264.150	2.000	1132.075	1.504	0.223
Face mask ✻ Distance ✻ Gender	761.288	2.000	380.644	0.506	0.603
Residual	317604.310	422.000	752.617		
Face mask ✻ Interaction	2.265	1.000	2.265	0.003	0.956
Face mask ✻ Interaction ✻ Gender	90.087	1.000	90.087	0.121	0.729
Residual	157354.118	211.000	745.754		
Distance ✻ Interaction	28280.045	2.000	14140.022	18.816	< .001
Distance ✻ Interaction ✻ Gender	77.976	2.000	38.988	0.052	0.949
Residual	317124.252	422.000	751.479		
Face mask ✻ Distance ✻ Interaction	28427.475	2.000	14213.737	18.329	< .001
Face mask ✻ Distance ✻ Interaction ✻ Gender	893.686	2.000	446.843	0.576	0.562
Residual	327255.413	422.000	775.487		

Note. Type III Sum of Squares

Supplementary Table 6

Results of the rmANOVA with the factor education

Within Subjects Effects

	**Sum of Squares**	**df**	**Mean Square**	**F**	**p**
Face mask	5383.967	1	5383.967	6.199	0.014
Face mask ✻ Education>4-Abi	9336.533	6	1556.089	1.792	0.102
Residual	181526.491	209	868.548		
Distance	18362.568	2	9181.284	10.779	< .001
Distance ✻ Education>4-Abi	8903.171	12	741.931	0.871	0.577
Residual	356026.232	418	851.737		
Interaction	5.460	1	5.460	0.006	0.936
Interaction ✻ Education>4-Abi	4308.743	6	718.124	0.842	0.539
Residual	178214.448	209	852.701		
Face mask ✻ Distance	4253.516	2	2126.758	2.910	0.056
Face mask ✻ Distance ✻ Education>4-Abi	14107.177	12	1175.598	1.608	0.086
Residual	305532.739	418	730.940		
Face mask ✻ Interaction	1339.400	1	1339.400	1.822	0.179
Face mask ✻ Interaction ✻ Education>4-Abi	3921.718	6	653.620	0.889	0.504
Residual	153653.884	209	735.186		
Distance ✻ Interaction	990.412	2	495.206	0.666	0.514
Distance ✻ Interaction ✻ Education>4-Abi	8488.920	12	707.410	0.951	0.495
Residual	310775.343	418	743.482		
Face mask ✻ Distance ✻ Interaction	4612.981	2	2306.491	2.971	0.052
Face mask ✻ Distance ✻ Interaction ✻ Education>4-Abi	4555.408	12	379.617	0.489	0.921
Residual	324526.095	418	776.378		

Note. Type III Sum of Squares

Supplementary Table 7

Results of the rmANCOVA with the covariate factor age

Within Subjects Effects

	Sum of Squares	df	Mean Square	F	p
Face mask	5412.331	1	5412.331	6.097	0.014
Face mask ✻ Age	882.125	1	882.125	0.994	0.320
Residual	189980.900	214	887.761		
Distance	3822.010	2	1911.005	2.242	0.108
Distance ✻ Age	62.474	2	31.237	0.037	0.964
Residual	364866.929	428	852.493		
Interaction	112.688	1	112.688	0.132	0.717
Interaction ✻ Age	19.277	1	19.277	0.023	0.881
Residual	182503.913	214	852.822		
Face mask ✻ Distance	424.237	2	212.118	0.284	0.753
Face mask ✻ Distance ✻ Age	41.560	2	20.780	0.028	0.973
Residual	319598.356	428	746.725		
Face mask ✻ Interaction	346.578	1	346.578	0.472	0.493
Face mask ✻ Interaction ✻ Age	431.945	1	431.945	0.588	0.444
Residual	157143.656	214	734.316		
Distance ✻ Interaction	6735.217	2	3367.608	4.545	0.011
Distance ✻ Interaction ✻ Age	2153.251	2	1076.626	1.453	0.235
Residual	317111.012	428	740.914		
Face mask ✻ Distance ✻ Interaction	1423.132	2	711.566	0.930	0.395
Face mask ✻ Distance ✻ Interaction ✻ Age	1548.587	2	774.294	1.012	0.364
Residual	327532.916	428	765.264		

Note. Type III Sum of Squares

Supplementary Table 8

Results of the rmANCOVA with the covariate factor Danger for Others

Within Subjects Effects

	Sum of Squares	df	Mean Square	F	p
Face mask	7.187	1	7.187	0.008	0.928
Face mask ✻ Covid_Danger_Others	750.160	1	750.160	0.844	0.359
Residual	190112.864	214	888.378		
Distance	8760.776	2	4380.388	5.177	0.006
Distance ✻ Covid_Danger_Others	2774.425	2	1387.213	1.639	0.195
Residual	362154.978	428	846.156		
Interaction	24.007	1	24.007	0.028	0.867
Interaction ✻ Covid_Danger_Others	25.490	1	25.490	0.030	0.863
Residual	182497.700	214	852.793		
Face mask ✻ Distance	159.921	2	79.961	0.107	0.898
Face mask ✻ Distance ✻ Covid_Danger_Others	60.964	2	30.482	0.041	0.960
Residual	319578.952	428	746.680		
Face mask ✻ Interaction	1703.997	1	1703.997	2.339	0.128
Face mask ✻ Interaction ✻ Covid_Danger_Others	1661.771	1	1661.771	2.281	0.132
Residual	155913.830	214	728.569		
Distance ✻ Interaction	169.087	2	84.544	0.114	0.893
Distance ✻ Interaction ✻ Covid_Danger_Others	910.154	2	455.077	0.612	0.543
Residual	318354.110	428	743.818		
Face mask ✻ Distance ✻ Interaction	7809.795	2	3904.898	5.120	0.006
Face mask ✻ Distance ✻ Interaction ✻ Covid_Danger_Others	2636.921	2	1318.460	1.729	0.179
Residual	326444.582	428	762.721		

Note. Type III Sum of Squares

Supplementary Table 9

R Results of the rmANCOVA with the covariate factor Danger for Young

Within Subjects Effects

	Sum of Squares	df	Mean Square	F	p
Face mask	5172.896	1	5172.896	5.811	0.017
Face mask ✻ Covid_Danger_Young	375.013	1	375.013	0.421	0.517
Residual	190488.012	214	890.131		
Distance	10930.951	2	5465.475	6.425	0.002
Distance ✻ Covid_Danger_Young	838.078	2	419.039	0.493	0.611
Residual	364091.325	428	850.681		
Interation	911.837	1	911.837	1.070	0.302
Interation ✻ Covid_Danger_Young	168.786	1	168.786	0.198	0.657
Residual	182354.405	214	852.123		
Face mask ✻ Distance	1374.430	2	687.215	0.921	0.399
Face mask ✻ Distance ✻ Covid_Danger_Young	328.592	2	164.296	0.220	0.802
Residual	319311.324	428	746.054		
Face mask ✻ Interation	718.058	1	718.058	0.979	0.323
Face mask ✻ Interation ✻ Covid_Danger_Young	677.322	1	677.322	0.924	0.338
Residual	156898.279	214	733.170		
Distance ✻ Interation	3411.235	2	1705.618	2.291	0.102
Distance ✻ Interation ✻ Covid_Danger_Young	645.415	2	322.708	0.433	0.649
Residual	318618.848	428	744.437		
Face mask ✻ Distance ✻ Interation	12349.708	2	6174.854	8.105	< .001
Face mask ✻ Distance ✻ Interation ✻ Covid_Danger_Young	2995.186	2	1497.593	1.966	0.141
Residual	326086.317	428	761.884		

Note. Type III Sum of Squares

Supplementary Table 10

Results of the rmANCOVA with the covariate factor Mask as Protection

Within Subjects Effects

	**Sum of Squares**	**df**	**Mean Square**	**F**	**p**
Face mask	2336.482	1	2336.482	2.621	0.107
Face mask ✻ Mask_Protection	83.695	1	83.695	0.094	0.760
Residual	190779.329	214	891.492		
Distance	4715.902	2	2357.951	2.775	0.063
Distance ✻ Mask_Protection	1226.857	2	613.429	0.722	0.486
Residual	363702.546	428	849.772		
Interaction	638.619	1	638.619	0.749	0.388
Interaction ✻ Mask_Protection	155.012	1	155.012	0.182	0.670
Residual	182368.178	214	852.188		
Face mask ✻ Distance	919.751	2	459.876	0.616	0.540
Face mask ✻ Distance ✻ Mask_Protection	341.705	2	170.853	0.229	0.795
Residual	319298.211	428	746.024		
Face mask ✻ Interaction	24.151	1	24.151	0.033	0.856
Face mask ✻ Interaction ✻ Mask_Protection	41.382	1	41.382	0.056	0.813
Residual	157534.219	214	736.141		
Distance ✻ Interaction	5833.986	2	2916.993	3.955	0.020
Distance ✻ Interaction ✻ Mask_Protection	3572.270	2	1786.135	2.422	0.090
Residual	315691.993	428	737.598		
Face mask ✻ Distance ✻ Interaction	4572.233	2	2286.117	2.988	0.051
Face mask ✻ Distance ✻ Interaction ✻ Mask_Protection	1639.735	2	819.868	1.072	0.343
Residual	327441.768	428	765.051		

Note. Type III Sum of Squares

Supplementary Table 11

Results of the rmANCOVA with the covariate factor Living with an Infected Person

Within Subjects Effects

	**Sum of Squares**	**df**	**Mean Square**	**F**	**p**
Face mask	162.545	1	162.545	0.183	0.670
Face mask ✻ Covid_Living_Infected	350.451	1	350.451	0.394	0.531
Residual	190512.574	214	890.246		
Distance	8463.298	2	4231.649	4.999	0.007
Distance ✻ Covid_Living_Infected	2637.908	2	1318.954	1.558	0.212
Residual	362291.495	428	846.475		
Interaction	859.864	1	859.864	1.010	0.316
Interaction ✻ Covid_Living_Infected	369.276	1	369.276	0.434	0.511
Residual	182153.915	214	851.187		
Face mask ✻ Distance	1531.303	2	765.652	1.028	0.359
Face mask ✻ Distance ✻ Covid_Living_Infected	869.938	2	434.969	0.584	0.558
Residual	318769.978	428	744.790		
Face mask ✻ Interaction	11.387	1	11.387	0.015	0.901
Face mask ✻ Interaction ✻ Covid_Living_Infected	5.380	1	5.380	0.007	0.932
Residual	157570.222	214	736.309		
Distance ✻ Interaction	546.328	2	273.164	0.368	0.692
Distance ✻ Interaction ✻ Covid_Living_Infected	1398.978	2	699.489	0.942	0.391
Residual	317865.286	428	742.676		
Face mask ✻ Distance ✻ Interaction	8048.203	2	4024.102	5.290	0.005
Face mask ✻ Distance ✻ Interaction ✻ Covid_Living_Infected	3499.373	2	1749.686	2.300	0.101
Residual	325582.130	428	760.706		

Note. Type III Sum of Squares

Supplementary Table 12

Results of the rmANCOVA with the covariate factor Living with a Person with Risk Factors

Within Subjects Effects

	Sum of Squares	df	Mean Square	F	p
Face mask	1411.468	1	1411.468	1.583	0.210
Face mask ✻ Covid_Mitwohnen_Risiko-quantised	44.122	1	44.122	0.049	0.824
Residual	190818.903	214	891.677		
Distance	223.057	2	111.529	0.132	0.876
Distance ✻ Covid_Living_Risk	3270.105	2	1635.052	1.935	0.146
Residual	361659.298	428	844.998		
Interaction	0.011	1	0.011	1.279e -5	0.997
Interaction ✻ Covid_Living_Risk	223.796	1	223.796	0.263	0.609
Residual	182299.395	214	851.866		
Face mask ✻ Distance	302.244	2	151.122	0.203	0.816
Face mask ✻ Distance ✻ Covid_Living_Risk	915.992	2	457.996	0.615	0.541
Residual	318723.924	428	744.682		
Face mask ✻ Interaction	618.329	1	618.329	0.844	0.359
Face mask ✻ Interaction ✻ Covid_Living_Risk	738.168	1	738.168	1.007	0.317
Residual	156837.433	214	732.885		
Distance ✻ Interaction	1513.908	2	756.954	1.018	0.362
Distance ✻ Interaction ✻ Covid_Living_Risk	934.885	2	467.443	0.628	0.534
Residual	318329.378	428	743.760		
Face mask ✻ Distance ✻ Interaction	4933.773	2	2466.886	3.217	0.041
Face mask ✻ Distance ✻ Interaction ✻ Covid_Living_Risk	894.498	2	447.249	0.583	0.559
Residual	328187.005	428	766.792		

Note. Type III Sum of Squares

Supplementary Table 13

Results of the rmANCOVA with the covariate factor Own Risk

Within Subjects Effects

	**Sum of Squares**	**df**	**Mean Square**	**F**	**p**
Face mask	4227.773	1	4227.773	4.749	0.030
Face mask ✻ Covid_Own_Risk	355.929	1	355.929	0.400	0.528
Residual	190507.096	214	890.220		
Distance	5187.172	2	2593.586	3.042	0.049
Distance ✻ Covid_Own_Risk	42.584	2	21.292	0.025	0.975
Residual	364886.819	428	852.539		
Interaction	209.602	1	209.602	0.246	0.621
Interaction ✻ Covid_Own_Risk	1.228	1	1.228	0.001	0.970
Residual	182521.962	214	852.906		
Face mask ✻ Distance	1069.087	2	534.543	0.717	0.489
Face mask ✻ Distance ✻ Covid_Own_Risk	362.817	2	181.408	0.243	0.784
Residual	319277.100	428	745.975		
Face mask ✻ Interaction	8.323	1	8.323	0.011	0.915
Face mask ✻ Interaction ✻ Covid_Own_Risk	1.681	1	1.681	0.002	0.962
Residual	157573.920	214	736.327		
Distance ✻ Interaction	5760.136	2	2880.068	3.910	0.021
Distance ✻ Interaction ✻ Covid_Own_Risk	4041.111	2	2020.555	2.743	0.065
Residual	315223.153	428	736.503		
Face mask ✻ Distance ✻ Interaction	5136.372	2	2568.186	3.342	0.036
Face mask ✻ Distance ✻ Interaction ✻ Covid_Own_Risk	152.280	2	76.140	0.099	0.906
Residual	328929.223	428	768.526		

Note. Type III Sum of Squares

Supplementary Table 14

Results of the rmANCOVA with the covariate factor Being worried about COVID-19

Within Subjects Effects

	Sum of Squares	df	Mean Square	F	p
Face mask	830.583	1	830.583	0.936	0.334
Face mask ✻ Covid_Fear	993.115	1	993.115	1.119	0.291
Residual	189869.910	214	887.243		
Distance	8071.273	2	4035.636	4.739	0.009
Distance ✻ Covid_Fear	490.533	2	245.266	0.288	0.750
Residual	364438.870	428	851.493		
Interaction	788.728	1	788.728	0.925	0.337
Interaction ✻ Covid_Fear	81.210	1	81.210	0.095	0.758
Residual	182441.981	214	852.533		
Face mask ✻ Distance	26.586	2	13.293	0.018	0.982
Face mask ✻ Distance ✻ Covid_Fear	304.240	2	152.120	0.204	0.816
Residual	319335.676	428	746.111		
Face mask ✻ Interaction	557.874	1	557.874	0.760	0.384
Face mask ✻ Interaction ✻ Covid_Fear	516.627	1	516.627	0.704	0.402
Residual	157058.975	214	733.920		
Distance ✻ Interaction	5126.952	2	2563.476	3.437	0.033
Distance ✻ Interaction ✻ Covid_Fear	69.890	2	34.945	0.047	0.954
Residual	319194.373	428	745.781		
Face mask ✻ Distance ✻ Interaction	9193.178	2	4596.589	5.984	0.003
Face mask ✻ Distance ✻ Interaction ✻ Covid_Fear	307.351	2	153.676	0.200	0.819
Residual	328774.152	428	768.164		

Note. Type III Sum of Squares

Supplementary Table 15

Results of the rmANCOVA with the covariate factor Danger of COVID-19

Within Subjects Effects

	**Sum of Squares**	**df**	**Mean Square**	**F**	**p**
Face mask	2114.254	1	2114.254	2.371	0.125
Face mask ✻ Covid_Danger	20.342	1	20.342	0.023	0.880
Residual	190842.682	214	891.788		
Distance	10033.342	2	5016.671	5.915	0.003
Distance ✻ Covid_Danger	1908.376	2	954.188	1.125	0.326
Residual	363021.027	428	848.180		
Interaction	615.428	1	615.428	0.722	0.396
Interaction ✻ Covid_Danger	70.272	1	70.272	0.082	0.774
Residual	182452.919	214	852.584		
Face mask ✻ Distance	1182.463	2	591.231	0.793	0.453
Face mask ✻ Distance ✻ Covid_Danger	398.575	2	199.288	0.267	0.766
Residual	319241.341	428	745.891		
Face mask ✻ Interaction	1185.580	1	1185.580	1.622	0.204
Face mask ✻ Interaction ✻ Covid_Danger	1151.779	1	1151.779	1.576	0.211
Residual	156423.823	214	730.952		
Distance ✻ Interaction	1114.444	2	557.222	0.750	0.473
Distance ✻ Interaction ✻ Covid_Danger	1080.153	2	540.076	0.726	0.484
Residual	318184.110	428	743.421		
Face mask ✻ Distance ✻ Interaction	17799.704	2	8899.852	11.792	< .001
Face mask ✻ Distance ✻ Interaction ✻ Covid_Danger	6053.362	2	3026.681	4.010	0.019
Residual	323028.142	428	754.739		

Note. Type III Sum of Squares

Supplementary Table 16

Results of the rmANCOVA with the covariate factor Danger for the Elderly

Within Subjects Effects

	**Sum of Squares**	**df**	**Mean Square**	**F**	**p**
Face mask	532.476	1	532.476	0.602	0.439
Face mask ✻ Covid_Elderly	1596.172	1	1596.172	1.805	0.181
Residual	189266.853	214	884.425		
Distance	1966.957	2	983.478	1.155	0.316
Distance ✻ Covid_Elderly	388.765	2	194.383	0.228	0.796
Residual	364540.638	428	851.730		
Interaction	68.828	1	68.828	0.081	0.777
Interaction ✻ Covid_Elderly	7.552	1	7.552	0.009	0.925
Residual	182515.638	214	852.877		
Face mask ✻ Distance	634.146	2	317.073	0.426	0.653
Face mask ✻ Distance ✻ Covid_Elderly	991.051	2	495.526	0.666	0.515
Residual	318648.865	428	744.507		
Face mask ✻ Interaction	3333.082	1	3333.082	4.623	0.033
Face mask ✻ Interaction ✻ Covid_Elderly	3292.612	1	3292.612	4.567	0.034
Residual	154282.990	214	720.949		
Distance ✻ Interaction	8.578	2	4.289	0.006	0.994
Distance ✻ Interaction ✻ Covid_Elderly	379.733	2	189.867	0.255	0.775
Residual	318884.530	428	745.057		
Face mask ✻ Distance ✻ Interaction	3236.296	2	1618.148	2.127	0.120
Face mask ✻ Distance ✻ Interaction ✻ Covid_Elderly	3417.476	2	1708.738	2.246	0.107
Residual	325664.027	428	760.897		

Note. Type III Sum of Squares

Supplementary Table 17

Results of the rmANCOVA with the covariate the factor Own Infection

Within Subjects Effects

	**Sum of Squares**	**df**	**Mean Square**	**F**	**p**
Face mask	2317.104	1	2317.104	2.599	0.108
Face mask ✻ Own_Infection	102.699	1	102.699	0.115	0.735
Residual	190760.326	214	891.403		
Distance	11550.354	2	5775.177	6.820	0.001
Distance ✻ Own_Infection	2508.723	2	1254.361	1.481	0.228
Residual	362420.680	428	846.777		
Interaction	2224.401	1	2224.401	2.626	0.107
Interaction Own_Infection	1239.123	1	1239.123	1.463	0.228
Residual	181284.068	214	847.122		
Face mask ✻ Distance	5593.536	2	2796.768	3.803	0.023
Face mask ✻ Distance ✻ Own_Infection	4910.640	2	2455.320	3.339	0.036
Residual	314729.277	428	735.349		
Face mask ✻ Interaction	476.469	1	476.469	0.649	0.421
Face mask ✻ Interaction ✻ Own_Infection	438.512	1	438.512	0.597	0.441
Residual	157137.089	214	734.285		
Distance ✻ Interaction	1543.345	2	771.672	1.035	0.356
Distance ✻ Interaction ✻ Covid_ Own_Infection	122.174	2	61.087	0.082	0.921
Residual	319142.090	428	745.659		
Face mask ✻ Distance ✻ Interaction	4069.760	2	2034.880	2.648	0.072
Face mask ✻ Distance ✻ Interaction ✻ Covid_Own_Infection	157.149	2	78.575	0.102	0.903
Residual	328924.354	428	768.515		

Note. Type III Sum of Squares

Supplementary Table 18

Results of the rmANOVA with the factor GAD-7

Within Subjects Effects

Cases	Sum of Squares	df	Mean Square	F	p
Interaction	3532.854	1	3532.854	4.151	0.043
Interaction ✻ GAD-7	384.389	1	384.389	0.452	0.502
Residuals	182138.801	214	851.116		
Distance	80201.851	2	40100.925	47.540	< .001
Distance ✻ GAD-7	3901.540	2	1950.770	2.313	0.100
Residuals	361027.863	428	843.523		
Mask	35621.399	1	35621.399	40.144	< .001
Mask ✻ GAD-7	971.468	1	971.468	1.095	0.297
Residuals	189891.557	214	887.344		
Interaction ✻ Distance	51540.308	2	25770.154	34.793	< .001
Interaction ✻ Distance ✻ GAD-7	2258.848	2	1129.424	1.525	0.219
Residuals	317005.415	428	740.667		
Interaction ✻ Mask	45.399	1	45.399	0.062	0.804
Interaction ✻ Mask ✻ GAD-7	6.328	1	6.328	0.009	0.926
Residuals	157569.273	214	736.305		
Distance ✻ Mask	4741.851	2	2370.926	3.175	0.043
Distance ✻ Mask ✻ GAD-7	16.800	2	8.400	0.011	0.989
Residuals	319623.117	428	746.783		
Interaction ✻ Distance ✻ Mask	62964.726	2	31482.363	40.969	< .001
Interaction ✻ Distance ✻ Mask ✻ GAD-7	189.586	2	94.793	0.123	0.884
Residuals	328891.918	428	768.439		

Note. Type III Sum of Squares

Supplementary Table 19

Anova results of the stimuli comparison in the valence variable

Factor	Sum of Squares	df	Mean Square	F	p
Mask	1.460	1	1.460	30.898	< .001
Distance	1.697	2	0.849	17.967	< .001
Mask ✻ Distance	0.244	2	0.122	2.579	0.090
Interaction	1.559	1	1.559	33.000	< .001
Mask ✻ Interaction	0.007	1	0.007	0.143	0.707
Distance ✻ Interaction	0.059	2	0.029	0.623	0.542
Mask ✻ Distance ✻ Interaction	0.024	2	0.012	0.258	0.774
Residuals	1.701	36	0.047		

Note. Type III Sum of Squares

Supplementary Table 20

Post-hoc tests results of the stimuli comparison in the valence variable as to the factor mask

		Mean Difference	SE	t	p_{holm}
No mask	Face mask	0.349	0.063	5.559	< .001 ***

*** p < .001

Note. Results are averaged over the levels of: Distance, Interaction

Supplementary Table 21

Post-hoc tests results of the stimuli comparison in the valence variable as to the factor distance

		Mean Difference	SE	t	p_{holm}
50	90	-0.039	0.077	-0.504	0.617
	150	0.378	0.077	4.921	< .001 ***
90	150	0.417	0.077	5.425	< .001 ***

*** p < .001

Note. P-value adjusted for comparing a family of 3

Note. Results are averaged over the levels of: Mask, Interaction

Supplementary Table 22

Post-hoc tests results of the stimuli comparison in the valence variable as to the factor interaction

		Mean Difference	SE	t	p_{holm}	
no	sh	-0.360	0.063	-5.745	< .001	***

*** p < .001

Note. Results are averaged over the levels of: Mask, Distance

Supplementary Table 23

Anova results of the stimuli comparison in the arousal variable

Cases	Sum of Squares	df	Mean Square	F	p
Mask	0.284	1	0.284	20.764	< .001
Distance	0.103	2	0.052	3.771	0.033
Mask ✻ Distance	0.140	2	0.070	5.129	0.011
Interaction	0.129	1	0.129	9.455	0.004
Mask ✻ Interaction	0.050	1	0.050	3.664	0.064
Distance ✻ Interaction	0.150	2	0.075	5.503	0.008
Mask ✻ Distance ✻ Interaction	0.015	2	0.007	0.535	0.590
Residuals	0.492	36	0.014		

Note. Type III Sum of Squares

Supplementary Table 24

Post-hoc tests results of the stimuli comparison in the arousal variable as to the factor mask

		Mean Difference	SE	t	p_{holm}	
nfm	wfm	-0.154	0.034	-4.557	< .001	***

*** p < .001

Note. Results are averaged over the levels of: Distance, Interaction

Supplementary Table 25

Post-hoc tests results of the stimuli comparison in the arousal variable as to the factor distance

		Mean Difference	SE	t	p_{holm}	
50	90	0.026	0.041	0.635	0.529	
	150	-0.082	0.041	-1.996	0.107	
90	150	-0.109	0.041	-2.632	0.037	*

* p < .05
Note. P-value adjusted for comparing a family of 3
Note. Results are averaged over the levels of: Mask, Interaction

Supplementary Table 26

Post-hoc tests results of the stimuli comparison in the arousal variable as to the factor interaction

		Mean Difference	SE	t	p_{holm}
no	sh	0.104	0.034	3.075	0.004 **

** p < .01
Note. Results are averaged over the levels of: Mask, Distance

Supplementary Table 27

*Post-hoc tests results of the stimuli comparison in the arousal variable as to the factors mask*distance*

		Mean Difference	SE	t	p_{holm}
nfm 50	wfm 50	-0.239	0.058	-4.085	0.003 **
	nfm 90	0.018	0.058	0.299	1.000
	wfm 90	-0.204	0.058	-3.486	0.013 *
	nfm 150	-0.201	0.058	-3.444	0.013 *
	wfm 150	-0.203	0.058	-3.465	0.013 *
wfm 50	nfm 90	0.256	0.058	4.385	0.001 **
	wfm 90	0.035	0.058	0.599	1.000
	nfm 150	0.038	0.058	0.642	1.000
	wfm 150	0.036	0.058	0.620	1.000
nfm 90	wfm 90	-0.221	0.058	-3.786	0.007 **
	nfm 150	-0.219	0.058	-3.743	0.007 **
	wfm 150	-0.220	0.058	-3.764	0.007 **
wfm 90	nfm 150	0.002	0.058	0.043	1.000
	wfm 150	0.001	0.058	0.021	1.000
nfm 150	wfm 150	-0.001	0.058	-0.021	1.000

* p < .05, ** p < .01
Note. P-value adjusted for comparing a family of 6
Note. Results are averaged over the levels of: Interaction

Supplementary Table 28

Post-hoc tests results of the stimuli comparison in the arousal variable as to the factor

*interaction*distance*

		Mean Difference	SE	t	p holm	
50 no	90 no	0.040	0.058	0.684	1.000	
	150 no	0.042	0.058	0.727	1.000	
	50 sh	0.196	0.058	3.358	0.022	*
	90 sh	0.209	0.058	3.572	0.014	*
	150 sh	-0.011	0.058	-0.192	1.000	
90 no	150 no	0.002	0.058	0.043	1.000	
	50 sh	0.156	0.058	2.674	0.101	
	90 sh	0.169	0.058	2.887	0.072	
	150 sh	-0.051	0.058	-0.877	1.000	
150 no	50 sh	0.154	0.058	2.631	0.101	
	90 sh	0.166	0.058	2.845	0.073	
	150 sh	-0.054	0.058	-0.920	1.000	
50 sh	90 sh	0.013	0.058	0.214	1.000	
	150 sh	-0.207	0.058	-3.551	0.014	*
90 sh	150 sh	-0.220	0.058	-3.764	0.009	**

* p < .05, ** p < .01

Note. P-value adjusted for comparing a family of 6

Note. Results are averaged over the levels of: Mask

Supplementary Table 29

Anova results of the stimuli comparison in the danger variable

Cases	Sum of Squares	df	Mean Square	F	p
Mask	0.585	1	0.585	22.679	< .001
Distance	0.178	2	0.089	3.451	0.043
Mask ∗ Distance	0.157	2	0.079	3.048	0.060
Interaction	0.150	1	0.150	5.799	0.021
Mask ∗ Interaction	0.065	1	0.065	2.501	0.123
Distance ∗ Interaction	0.109	2	0.055	2.112	0.136
Mask ∗ Distance ∗ Interaction	0.048	2	0.024	0.930	0.404
Residuals	0.929	36	0.026		

Note. Type III Sum of Squares

Supplementary Table 30

Post-hoc tests results of the stimuli comparison in the danger variable as to the factor mask

		Mean Difference	SE	t	p_{holm}
nfm	wfm	-0.221	0.046	-4.762	< .001 ***

*** p < .001
Note. Results are averaged over the levels of: Distance, Interaction

Supplementary Table 31

Post-hoc tests results of the stimuli comparison in the danger variable as to the factor distance

		Mean Difference	SE	t	p_{holm}
50	90	0.099	0.057	1.739	0.181
	150	-0.047	0.057	-0.836	0.408
90	150	-0.146	0.057	-2.575	0.043 *

* p < .05
Note. P-value adjusted for comparing a family of 3
Note. Results are averaged over the levels of: Mask, Interaction

Supplementary Table 32

Summary of the linear mixed model with random intercept for factors ID and Stimuli

**Random effects**	**Name**	**Variance**	**SD**	
ID	(Intercept)	4116.3	64.16	
Stimuli	(Intercept)	304.3	17.44	
Residual		3321.6	57.63	
**Fixed effects**	***b***	***SE***	***t***	***p***
(Intercept)	631.769 **	5.118	123.446	**<0.001**
Distance2-1	-8.276	6.381	-1.297	0.195
Distance3-2	14.650*	6.384	2.295	**0.022**
InteractionD.sum_int	2.372	5.217	0.455	0.649
MaskD.sum_mask	-7.315	5.216	-1.402	0.161
Distance2-1:InteractionD.sum_int	10.459	12.760	0.820	0.412
Distance3-2:InteractionD.sum_int	-22.288	12.766	-1.746	0.081
Distance2-1:MaskD.sum_mask	-1.166	12.759	-0.091	0.927

Distance3-2:MaskD.sum_mask	8.077	12.764	0.633	0.527
InteractionD.sum_int * MaskD.sum_mask	-0.958	10.425	-0.092	0.927
Distance2-1:InteractionD.sum_int:MaskD.sum_mask	-19.602	25.518	-0.768	0.442
Distance3-2:InteractionD.sum_int:MaskD.sum_mask	-28.665	25.528	-1.123	0.261
Random Effects				
σ2	3321.593			
τ00 ID	4116.270			
τ00 Stimuli	304.279			
ICC	0.571			
N ID	213			
N Stimuli	48			
Observations	7459			
Marginal R2 / Conditional R2	0.013 / 0.576			

* $p<0.05$ ** $p<0.01$
